# Supplementary figures and images for: The number of cases, mortality and treatments of viral hemorrhagic fevers: A systematic review
Source: PLoS Negl Trop Dis. 2022 Oct 31;16(10):e0010889. doi: 10.1371/journal.pntd.0010889 (PMC9648854; doi:10.1371/journal.pntd.0010889)

S1 Fig. MEDLINE Search strategy


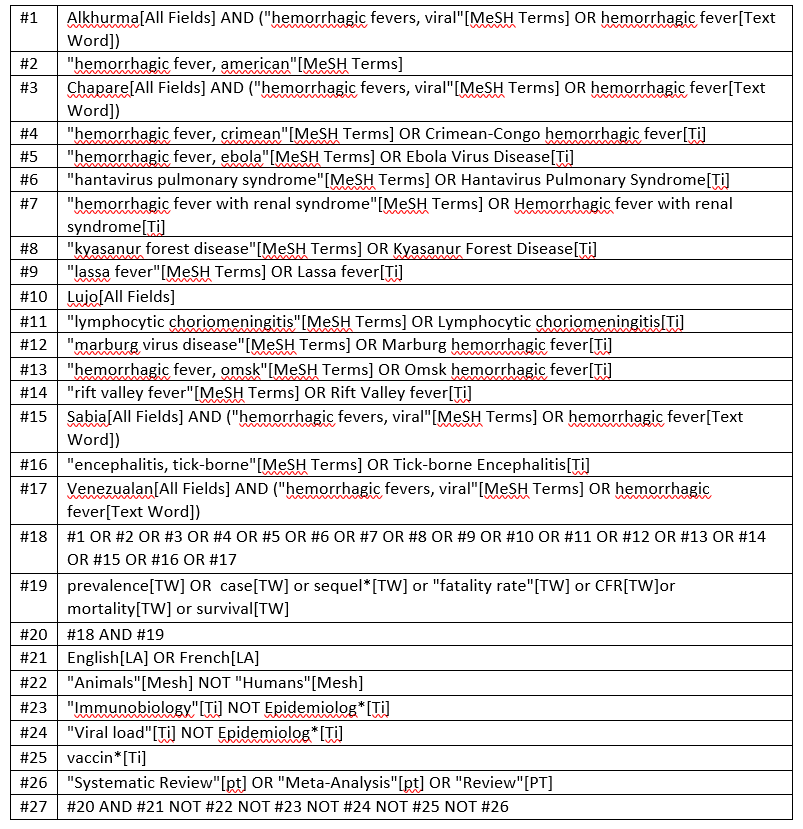

Supplement: S1 Fig — (DOCX) [file pntd.0010889.s001.docx]
