# Supplementary material for: The number of cases, mortality and treatments of viral hemorrhagic fevers: A systematic review
Source: PLoS Negl Trop Dis. 2022 Oct 31;16(10):e0010889. doi: 10.1371/journal.pntd.0010889 (PMC9648854; doi:10.1371/journal.pntd.0010889)
Supplement: S1 Table — (DOCX) [file pntd.0010889.s002.docx]

S1 Table. References details of each included publication.

| **Study focus** | **VHF** | **Study number** | **First author name** | **Year** | **Title** | **Journal** | **Pages** | **Issue** | **Volume** | **DOI** |
| --- | --- | --- | --- | --- | --- | --- | --- | --- | --- | --- |
| Cases/CFR | EVD | 6 |  | 2014 | Update: Ebola virus disease outbreak--West Africa, October 2014 | MMWR. Morbidity and mortality weekly report | 978-981 | 43 | 63 |  |
| Cases/CFR | EVD | 7 |  | 2014 | Update: Ebola virus disease epidemic--West Africa, November 2014 | MMWR. Morbidity and mortality weekly report | 1064-1066 | 46 | 63 |  |
| Cases/CFR | EVD | 8 |  | 2014 | Update: ebola virus disease epidemic - West Africa, December 2014 | MMWR. Morbidity and mortality weekly report | 1199-1201 | 50 | 63 |  |
| Cases/CFR | EVD | 21 |  | 1996 | Outbreak of Ebola haemorrhagic fever in Gabon | Communicable disease report. CDR weekly | 75-78 | 9 | 6 |  |
| Cases/CFR | CCHF | 31 |  | 2006 | Increase in cases of Crimean-Congo haemorrhagic fever, Turkey, 2006 | Euro surveillance : bulletin Europeen sur les maladies transmissibles = European communicable disease bulletin | E060720.2-E060720.2 | 7 | 11 |  |
| Cases/CFR | EVD | 53 |  | 2014 | Ebola virus disease outbreak - West Africa, September 2014 | MMWR. Morbidity and mortality weekly report | 865-866 | 39 | 63 |  |
| Cases/CFR | EVD | 54 |  | 2015 | Ebola virus disease (EVD) in West Africa: an extraordinary epidemic | Releve epidemiologique hebdomadaire | 89-96 | 10 | 90 |  |
| Cases/CFR | EVD | 56 |  | 1997 | Ebola haemorrhagic fever. A summary of the outbreak in Gabon | Releve epidemiologique hebdomadaire | 44050 | 43862 | 72 |  |
| Cases/CFR | RVF | 66 |  | 1998 | An outbreak of Rift Valley Fever, eastern Africa, 1997-1998 | Releve epidemiologique hebdomadaire | 105-109 | 12 | 73 |  |
| Cases/CFR | CCHF | 119 | Duran | 2013 | Evaluation of patients with Crimean-Congo hemorrhagic fever in Bolu, Turkey | African Health Sciences | 233-242 | 2 | 13 | 10.4314/ahs.v13i2.5 |
| Cases/CFR | RVF | 163 | Lagare | 2019 | First occurrence of Rift Valley fever outbreak in Niger, 2016 | Veterinary medicine and science | 70-78 | 1 | 5 | 10.1002/vms3.135 |
| Cases/CFR | EVD | 210 | Rosello | 2015 | Ebola virus disease in the Democratic Republic of the Congo,1976-2014 | eLife | e09015-e09015 | NOVEMBER2015 | 4 | 10.7554/eLife.09015 |
| Cases/CFR | CCHF | 239 | Tumturk | 2019 | Crimean-Congo haemorrhagic fever in a middle Anatolian city: five years of experience | Tropical Doctor |  |  |  | 10.1177/0049475519891337 |
| Cases/CFR | CCHF | 252 | Grobbelaar | 2014 | Epidemiologic and phylogenetic characteristics of Crimean-Congo haemorrhagic fever in South Africa, 1981-2013 | International Journal of Infectious Diseases | 191-191 |  | 21 | 10.1016/j.ijid.2014.03.818 |
| Treatment | EVD | 313 | Aluisio | 2019 | Association between vitamin a supplementation and mortality in Ebola virus disease: A multisite cohort study | Academic Emergency Medicine | S67-S67 |  | 26 | 10.1111/acem.13756 |
| Treatment | EVD | 315 | Aluisio | 2019 | Impact of Intravenous Fluid Therapy on Survival Among Patients with Ebola Virus Disease: An International Multisite Retrospective Cohort Study | Clinical infectious diseases : an official publication of the Infectious Diseases Society of America | ciz344-ciz344 |  |  | 10.1093/cid/ciz344 |
| Cases/CFR | BHF | 361 | Aguilar | 2009 | Reemergence of Bolivian hemorrhagic fever, 2007-2008 | Emerging infectious diseases | 1526-1528 | 9 | 15 | 10.3201/eid1509.090017 |
| Cases/CFR | CCHF | 383 | Al-Abri | 2019 | Clinical and molecular epidemiology of Crimean-Congo hemorrhagic fever in Oman | PLoS neglected tropical diseases | e0007100-e0007100 | 4 | 13 | 10.1371/journal.pntd.0007100 |
| Cases/CFR | HPS | 403 | Alonso | 2019 | Epidemiological description, case-fatality rate, and trends of Hantavirus Pulmonary Syndrome: 9 years of surveillance in Argentina | Journal of medical virology | 1173-1181 | 7 | 91 | 10.1002/jmv.25446 |
| Treatment | EVD | 408 | Aluisio | 2019 | Vitamin A Supplementation Was Associated with Reduced Mortality in Patients with Ebola Virus Disease during the West African Outbreak | Journal of Nutrition | 1757-1765 | 10 | 149 | 10.1093/jn/nxz142 |
| Treatment | EVD | 409 | Aluisio | 2020 | Association Between Treatment with Oral Third-Generation Cephalosporin Antibiotics and Mortality Outcomes in Ebola Virus Disease: A Multinational Retrospective Cohort Study | Tropical medicine & international health : TM & IH | 10.1111/tmi.13369-10.1111/tmi.13369 |  |  | 10.1111/tmi.13369 |
| Cases/CFR | RVF | 432 | Archer | 2013 | Epidemiologic Investigations into Outbreaks of Rift Valley Fever in Humans, South Africa, 2008-2011 | Emerging infectious diseases | 1918-1925 | 12 | 19 | 10.3201/eid1912.121527 |
| Cases/CFR | EVD | 469 | Aylward | 2014 | Ebola virus disease in West Africa--the first 9 months of the epidemic and forward projections | The New England journal of medicine | 1481-1495 | 16 | 371 | 10.1056/NEJMoa1411100 |
| Treatment | CCHF | 478 | Dokuzoguz | 2013 | Severity scoring index for Crimean-Congo hemorrhagic fever and the impact of ribavirin and corticosteroids on fatality | Clinical infectious diseases : an official publication of the Infectious Diseases Society of America | 1270-1274 | 9 | 57 | 10.1093/cid/cit527 |
| Cases/CFR | CCHF | 491 | Knust | 2011 | Crimean congo hemorrhagic fever surveillance in Kazakhstan, 2009-2010 | American Journal of Tropical Medicine and Hygiene | 282-282 | 6 | 85 |  |
| Cases/CFR | EVD | 646 | Bosch | 2004 | Sudan Ebola outbreak of known strain | The Lancet. Infectious diseases | 388-388 | 7 | 4 | 10.1016/s1473-3099(04)01071-0 |
| Cases/CFR | EVD | 698 | Butler | 2014 | Ebola by the numbers: The size, spread and cost of an outbreak | Nature | 284-285 | 7522 | 514 | 10.1038/514284a |
| Cases/CFR | EVD | 747 | Nanclares | 2016 | Ebola Virus Disease, Democratic Republic of the Congo, 2014 | Emerging infectious diseases | 1579-1586 | 9 | 22 | 10.3201/eid2209.160354 |
| Treatment | EVD | 804 | Bai | 2016 | Clinical and Virological Characteristics of Ebola Virus Disease Patients Treated with Favipiravir (T-705) - Sierra Leone, 2014 | Clinical infectious diseases : an official publication of the Infectious Diseases Society of America | 1288-1294 | 10 | 63 | 10.1093/cid/ciw571 |
| Treatment | CCHF | 852 | Cevik | 2008 | A preliminary study to evaluate the effect of intravenous ribavirin treatment on survival rates in Crimean-Congo hemorrhagic fever | The Journal of infection | 350-351 | 4 | 57 | 10.1016/j.jinf.2008.07.007 |
| Treatment | HPS | 862 | Chapman | 1999 | Intravenous ribavirin for hantavirus pulmonary syndrome: safety and tolerance during 1 year of open-label experience. Ribavirin Study Group | Antiviral Therapy | 211-219 | 4 | 4 |  |
| Cases/CFR | EVD | 878 | Chérif | 2017 | Ebola virus disease in children during the 2014–2015 epidemic in Guinea: a nationwide cohort study | European Journal of Pediatrics | 791-796 | 6 | 176 | 10.1007/s00431-017-2914-z |
| Cases/CFR | CCHF | 887 | Chinikar | 2010 | Geographical distribution and surveillance of Crimean-Congo hemorrhagic fever in Iran | Vector-Borne and Zoonotic Diseases | 705-708 | 7 | 10 | 10.1089/vbz.2009.0247 |
| Treatment | EVD | 1005 | Sissoko | 2016 | Experimental Treatment with Favipiravir for Ebola Virus Disease (the JIKI Trial): A Historically Controlled, Single-Arm Proof-of-Concept Trial in Guinea | PLoS Medicine | e1001967-e1001967 | 3 | 13 | 10.1371/journal.pmed.1001967 |
| Treatment | EVD | 1014 | Yam | 2020 | Association between multivitamin supplementation and mortality among patients with Ebola virus disease: An international multisite cohort study | African Journal of Emergency Medicine |  |  |  | 10.1016/j.afjem.2019.11.001 |
| Cases/CFR | HPS | 1061 | da Rosa Elkhoury | 2012 | Hantavirus pulmonary syndrome: prognostic factors for death in reported cases in Brazil | Transactions of the Royal Society of Tropical Medicine and Hygiene | 298-302 | 5 | 106 | 10.1016/j.trstmh.2012.01.002 |
| Cases/CFR | LF | 1071 | Dan-Nwafor | 2019 | Measures to control protracted large Lassa fever outbreak in Nigeria, 1 January to 28 April 2019 | Eurosurveillance | 1900272-1900272 | 20 | 24 | 10.2807/1560-7917.ES.2019.24.20.1900272 |
| Cases/CFR | EVD | 1119 | Dixon | 2014 | Ebola viral disease outbreak--West Africa, 2014. | MMWR. Morbidity and mortality weekly report | 548-551 | 25 | 63 |  |
| Treatment | EVD | 1158 | Dunning | 2016 | Experimental Treatment of Ebola Virus Disease with Brincidofovir | PLoS ONE | e0162199-e0162199 | 9 | 11 | 10.1371/journal.pone.0162199 |
| Treatment | EVD | 1188 | Gignoux | 2016 | Effect of Artesunate-Amodiaquine on Mortality Related to Ebola Virus Disease | The New England journal of medicine | 23-32 | 1 | 374 | 10.1056/NEJMoa1504605 |
| Cases/CFR | HFRS | 1232 | Tkachenko | 2019 | Hemorrhagic fever with renal syndrome, Russia | Emerging infectious diseases | 2325-2328 | 12 | 25 | 10.3201/eid2512.181649 |
| Cases/CFR | EVD | 1235 | Adedire | 2015 | Descriptive epidemiology of the EBOLA virus disease outbreak in Nigeria, July to September 2014 | American Journal of Tropical Medicine and Hygiene | 55-56 | 4 | 93 |  |
| Treatment | CCHF | 1284 | Elaldi | 2009 | Efficacy of oral ribavirin treatment in Crimean-Congo haemorrhagic fever: a quasi-experimental study from Turkey | The Journal of infection | 238-244 | 3 | 58 | 10.1016/j.jinf.2009.01.014 |
| Cases/CFR | EVD | 1354 | Kateh | 2015 | Rapid response to Ebola outbreaks in remote areas - Liberia, July-November 2014 | MMWR. Morbidity and mortality weekly report | 188-192 | 7 | 64 |  |
| Treatment | EVD | 1361 | Sahr | 2017 | Evaluation of convalescent whole blood for treating Ebola Virus Disease in Freetown, Sierra Leone | The Journal of infection | 302-309 | 3 | 74 | 10.1016/j.jinf.2016.11.009 |
| Cases/CFR | CCHF | 1379 | Vescio | 2012 | Environmental correlates of Crimean-Congo haemorrhagic fever incidence in Bulgaria | BMC public health | 1116-1116 |  | 12 | 10.1186/1471-2458-12-1116 |
| Treatment | CCHF | 1506 | Yilmaz | 2016 | Ribavirin in treatment of crimean-congo hemorrhagic fever (CCHF): An international multicenter retrospective analysis | Open Forum Infectious Diseases |  |  | 3 | 10.1093/ofid/ofw172.531 |
| Treatment | HPS | 1512 | Mertz | 2004 | Placebo-controlled, double-blind trial of intravenous ribavirin for the treatment of hantavirus cardiopulmonary syndrome in North America | Clinical infectious diseases : an official publication of the Infectious Diseases Society of America | 1307-1313 | 9 | 39 | 10.1086/425007 |
| Cases/CFR | CCHF | 1521 | Yilmaz | 2009 | The epidemiology of Crimean-Congo hemorrhagic fever in Turkey, 2002-2007 | International journal of infectious diseases : IJID : official publication of the International Society for Infectious Diseases | 380-386 | 3 | 13 | 10.1016/j.ijid.2008.07.021 |
| Treatment | EVD | 1538 | Garbern | 2019 | Effect of Mass Artesunate-Amodiaquine Distribution on Mortality of Patients With Ebola Virus Disease During West African Outbreak | Open forum infectious diseases | ofz250-ofz250 | 7 | 6 | 10.1093/ofid/ofz250 |
| Treatment | HFRS | 1611 | Gui | 1987 | Hemorrhagic fever with renal syndrome: treatment with recombinant alpha interferon | Journal of infectious diseases | 1047-1051 | 5 | 155 | 10.1093/infdis/155.5.1047 |
| Treatment | HFRS | 1643 | Du | 2013 | The optimal timing of RRT for critical patients with hemorrhagic fever with renal syndrome | Therapeutic Apheresis and Dialysis | A2-A2 | 5 | 17 | 10.1111/1744-9987.12117 |
| Cases/CFR | HFRS | 1703 | Chen | 1993 | Epidemiologic surveillance on the hemorrhagic fever with renal syndrome in China | Chinese medical journal | 857-863 | 11 | 106 |  |
| Treatment | HFRS | 1800 | Huggins | 1991 | Prospective, double-blind, concurrent, placebo-controlled clinical trial of intravenous ribavirin therapy of hemorrhagic fever with renal syndrome | The Journal of infectious diseases | 1119-1127 | 6 | 164 | 10.1093/infdis/164.6.1119 |
| Cases/CFR | EVD | 1837 | Deng | 1978 | Ebola haemorrhagic fever in Sudan, 1976. Report of a WHO/International Study Team | Bulletin of the World Health Organization | 247-270 | 2 | 56 |  |
| Treatment | LF | 1850 | Ilori | 2019 | Epidemiologic and Clinical Features of Lassa Fever Outbreak in Nigeria, January 1-May 6, 2018 | Emerging infectious diseases | 1066-1074 | 6 | 25 | 10.3201/eid2506.181035 |
| Treatment | CCHF | 1863 | Izadi | 2009 | Evaluation of the efficacy of ribavirin therapy on survival of Crimean-Congo hemorrhagic fever patients: A case-control study | Japanese journal of infectious diseases | 42309 | 1 | 62 |  |
| Treatment | EVD | 1888 | Dunning | 2016 | Experimental Treatment of Ebola Virus Disease with TKM-130803: A Single-Arm Phase 2 Clinical Trial | PLoS Medicine | e1001997-e1001997 | 4 | 13 | 10.1371/journal.pmed.1001997 |
| Cases/CFR | LF | 1978 | Bangura | 2009 | Epidemiology of lassa fever in the mano river union countries of West Africa, 2004-2008 | American Journal of Tropical Medicine and Hygiene | 209-210 | 5 | 81 |  |
| Cases/CFR | HPS | 2209 | Khan | 1996 | Hantavirus pulmonary syndrome: The first 100 US cases | The Journal of infectious diseases | 1297-1303 | 6 | 173 | 10.1093/infdis/173.6.1297 |
| Cases/CFR | EVD | 2211 | Khan | 1999 | The reemergence of Ebola hemorrhagic fever, Democratic Republic of the Congo, 1995 | The Journal of infectious diseases | S76-S86 | SUPPL. 1 | 179 Suppl | 10.1086/514306 |
| Cases/CFR | HFRS | 2241 | Klein | 2011 | Sex differences in the incidence and case fatality rates from hemorrhagic fever with renal syndrome in China, 2004-2008 | Clinical infectious diseases : an official publication of the Infectious Diseases Society of America | 1414-1421 | 12 | 52 | 10.1093/cid/cir232 |
| Treatment | CCHF | 2258 | Koksal | 2010 | The efficacy of ribavirin in the treatment of Crimean-Congo hemorrhagic fever in Eastern Black Sea region in Turkey | Journal of Clinical Virology | 65-68 | 1 | 47 | 10.1016/j.jcv.2009.11.007 |
| Treatment | EVD | 2261 | Konde | 2017 | Interferon β-1a for the treatment of Ebola virus disease: A historically controlled, single-arm proof-of-concept trial | PLoS ONE | e0169255-e0169255 | 2 | 12 | 10.1371/journal.pone.0169255 |
| Cases/CFR | EVD | 2292 | Kucharski | 2014 | Case fatality rate for Ebola virus disease in West Africa | Lancet (London, England) | 1260-1260 | 9950 | 384 | 10.1016/S0140-6736(14)61706-2 |
| Cases/CFR | HFRS | 2434 | Lee | 2013 | Epidemiology of hemorrhagic fever with renal syndrome in Korea, 2001-2010 | Journal of Korean medical science | 1552-1554 | 10 | 28 | 10.3346/jkms.2013.28.10.1552 |
| Cases/CFR | HFRS | 2482 | Liu | 2019 | Long-term retrospective observation reveals stabilities and variations of hantavirus infection in Hebei, China | BMC infectious diseases | 765-765 | 1 | 19 | 10.1186/s12879-019-4402-8 |
| Cases/CFR | RVF | 2601 | Mohamed | 2010 | Epidemiologic and clinical aspects of a Rift Valley fever outbreak in humans in Tanzania, 2007 | The American journal of tropical medicine and hygiene | 22-27 | 2 Suppl | 83 | 10.4269/ajtmh.2010.09-0318 |
| Cases/CFR | HFRS | 2626 | Ratkovic | 2016 | Winter time hemorrhagic fever with renal syndrome in Montenegro-new challenge | Nephrology Dialysis Transplantation | i153-i154 |  | 31 | 10.1093/ndt/gfw162.21 |
| Cases/CFR | LF | 2692 | Buba | 2018 | Mortality Among Confirmed Lassa Fever Cases During the 2015-2016 Outbreak in Nigeria | American journal of public health | 262-264 | 2 | 108 | 10.2105/AJPH.2017.304186 |
| Cases/CFR | RVF | 2711 | Baba | 2014 | Cyclical outbreaks of rift valley fever in East Africa: Why they persist and possible solutions to prevent or contain its spread | American Journal of Tropical Medicine and Hygiene | 260-260 | 5 | 91 |  |
| Cases/CFR | RVF | 2712 | Baba | 2015 | Has rift valley fever virus evolved to increased virulence in human population after existence for a century in east Africa? | American Journal of Tropical Medicine and Hygiene | 201-201 | 4 | 93 |  |
| Cases/CFR | CCHF | 2718 | Sahak | 2019 | Descriptive epidemiology of Crimean-Congo Hemorrhagic Fever (CCHF) in Afghanistan: Reported cases to National Surveillance System, 2016–2018 | International journal of infectious diseases : IJID : official publication of the International Society for Infectious Diseases | 135-140 |  | 88 | 10.1016/j.ijid.2019.08.016 |
| Cases + Sequelaes | Multi (Argentine, Lassa, Lujo, LCM, Venezuelan) | 2728 | Wilson | 2014 | Diseases of the central nervous system caused by lymphocytic choriomeningitis virus and other arenaviruses | Handbook of Clinical Neurology | 671-681 |  | 123 | 10.1016/B978-0-444-53488-0.00033-X |
| Cases/CFR | EVD | 2752 | MacNeil | 2010 | Proportion of deaths and clinical features in Bundibugyo Ebola virus infection, Uganda | Emerging infectious diseases | 1969-1972 | 12 | 16 | 10.3201/eid1612.100627 |
| Cases/CFR | HPS | 2753 | MacNeil | 2011 | Hantavirus pulmonary syndrome, United States, 1993-2009 | Emerging infectious diseases | 1195-1201 | 7 | 17 | 10.3201/eid1707.101306 |
| Cases/CFR | AHF | 2755 | Madani | 2011 | Alkhumra (Alkhurma) virus outbreak in Najran, Saudi Arabia: epidemiological, clinical, and laboratory characteristics | The Journal of infection | 67-76 | 1 | 62 | 10.1016/j.jinf.2010.09.032 |
| Treatment | ArHF | 2766 | Maiztegui | 1979 | Efficacy of immune plasma in treatment of Argentine haemorrhagic fever and association between treatment and a late neurological syndrome | Lancet (London, England) | 1216-1217 | 8154 | 2 | 10.1016/s0140-6736(79)92335-3 |
| Cases/CFR | CCHF | 2767 | Majeed | 2012 | Morbidity and mortality of Crimean-Congo hemorrhagic fever in Iraq: cases reported to the National Surveillance System, 1990-2010 | Transactions of the Royal Society of Tropical Medicine and Hygiene | 480-483 | 8 | 106 | 10.1016/j.trstmh.2012.04.006 |
| Cases/CFR | HFRS | 2768 | Makary | 2010 | Disease burden of Puumala virus infections, 1995-2008 | Epidemiology and infection | 1484-1492 | 10 | 138 | 10.1017/S0950268810000087 |
| Treatment | CCHF | 2776 | Mardani | 2003 | The efficacy of oral ribavirin in the treatment of crimean-congo hemorrhagic fever in Iran | Clinical infectious diseases : an official publication of the Infectious Diseases Society of America | 1613-1618 | 12 | 36 | 10.1086/375058 |
| Treatment | LF | 2812 | McCormick | 1986 | Lassa fever: Effective therapy with ribavirin | The New England journal of medicine | 20-26 | 1 | 314 | 10.1056/NEJM198601023140104 |
| Cases/CFR | AHF | 2836 | Memish | 2014 | Is the epidemiology of alkhurma hemorrhagic fever changing?: A three-year overview in Saudi Arabia | PLoS ONE | e85564-e85564 | 2 | 9 | 10.1371/journal.pone.0085564 |
| Cases/CFR | CCHF | 2870 | Mofleh | 2012 | Crimean-congo haemorrhagic fever outbreak investigation in the western region of Afghanistan in 2008 | Eastern Mediterranean health journal = La revue de sante de la Mediterranee orientale = al-Majallah al-sihhiyah li-sharq al-mutawassit | 522-526 | 5 | 18 | 10.26719/2012.18.5.522 |
| Treatment | EVD | 2914 | Mulangu | 2019 | A Randomized, Controlled Trial of Ebola Virus Disease Therapeutics | The New England journal of medicine | 2293-2303 | 24 | 381 | 10.1056/NEJMoa1910993 |
| Cases/CFR | HFRS | 2915 | Mulić | 2002 | Epidemiologic characteristics and military implications of hemorrhagic fever with renal syndrome in Croatia | Croatian Medical Journal | 581-586 | 5 | 43 |  |
| Treatment | CCHF | 2961 | Tulek | 2012 | The evaluation of ribavirin use in patients with Crimean-Congo haemorrhagic fever | Clinical Microbiology and Infection | 579-580 |  | 18 | 10.1111/j.1469-0691.2012.03802.x |
| Cases/CFR | RVF | 3006 | Nguku | 2010 | An investigation of a major outbreak of rift valley fever in Kenya: 2006-2007 | The American journal of tropical medicine and hygiene | 41395 | 2 Suppl | 83 | 10.4269/ajtmh.2010.09-0288 |
| Cases/CFR | EVD | 3022 | Nkoghe | 2005 | Multiple Ebola virus haemorrhagic fever outbreaks in Gabon, from October 2001 to April 2002 | Bulletin de la Societe de pathologie exotique (1990) | 224-229 | 3 | 98 |  |
| Cases/CFR | EVD | 3096 | Okware | 2002 | An outbreak of Ebola in Uganda | Tropical medicine & international health : TM & IH | 1068-1075 | 12 | 7 | 10.1046/j.1365-3156.2002.00944.x |
| Cases/CFR | MVD | 3118 | Organization, World Health | 2005 | Marburg haemorrhagic fever, Angola--update | Releve epidemiologique hebdomadaire | 125-126 | 16 | 80 |  |
| Cases/CFR | CCHF | 3165 | Nabeth | 2004 | Crimean-Congo hemorrhagic fever, Mauritania | Emerging infectious diseases | 2143-2149 | 12 | 10 | 10.3201/eid1012.040535 |
| Treatment | HPS | 3197 | Vial | 2015 | A non-randomized multicentre trial of human immune plasma for treatment of hantavirus cardiopulmonary syndrome caused by Andes virus | Antiviral Therapy | 377-386 | 4 | 20 | 10.3851/IMP2875 |
| Treatment | HPS | 3198 | Vial | 2013 | High-dose intravenous methylprednisolone for hantavirus cardiopulmonary syndrome in Chile: A double-blind, randomized controlled clinical trial | Clinical infectious diseases : an official publication of the Infectious Diseases Society of America | 943-951 | 7 | 57 | 10.1093/cid/cit394 |
| Treatment | EVD | 3385 | Kerber | 2019 | Laboratory Findings, Compassionate Use of Favipiravir, and Outcome in Patients with Ebola Virus Disease, Guinea, 2015 - A Retrospective Observational Study | The Journal of infectious diseases | 195-202 | 2 | 220 | 10.1093/infdis/jiz078 |
| Treatment | EVD | 3424 | Sadek | 1999 | Ebola hemorrhagic fever, Democratic Republic of the Congo, 1995: determinants of survival | The Journal of infectious diseases | S24-S27 | SUPPL. 1 | 179 | 10.1086/514311 |
| Treatment | EVD | 3453 | Davey | 2016 | A Randomized, Controlled Trial of ZMapp for Ebola Virus Infection | New England journal of medicine | 1448-1456 | 15 | 375 | 10.1056/NEJMoa1604330 |
| Cases/CFR | HPS | 3507 | Riquelme | 2015 | Hantavirus pulmonary syndrome, southern chile, 1995–2012 | Emerging infectious diseases | 562-568 | 4 | 21 | 10.3201/eid2104.141437 |
| Cases/CFR | CCHF | 3565 | Chinikar | 2012 | Last situation of Crimean-Congo haemorrhagic fever in Iran and its public health importance | Clinical Microbiology and Infection | 716-716 |  | 18 | 10.1111/j.1469-0691.2012.03803.x |
| Cases/CFR | HFRS | 3654 | Zhang | 2014 | Epidemic characteristics of hemorrhagic fever with renal syndrome in China, 2006-2012 | BMC infectious diseases | 384-384 | 1 | 14 | 10.1186/1471-2334-14-384 |
| Cases/CFR | CCHF | 3698 | Tabatabaei | 2014 | Crimean Congo hemorrhagic fever case fatality and associated factors in southeast of Iran, 1999-2012 | International Journal of Infectious Diseases | 240-240 |  | 21 | 10.1016/j.ijid.2014.03.921 |
| Treatment | CCHF | 3733 | Salehi | 2013 | Comparative study between Ribavirin and Ribavirin plus Intravenous Immunoglobulin against Crimean Congo hemorrhagic fever | Journal of Research in Medical Sciences | 497-500 | 6 | 18 |  |
| Cases/CFR | CCHF | 3983 | Nurmakhanov | 2015 | Crimean-Congo haemorrhagic fever virus in Kazakhstan (1948-2013) | International journal of infectious diseases : IJID : official publication of the International Society for Infectious Diseases | 19-23 |  | 38 | 10.1016/j.ijid.2015.07.007 |
| Cases/CFR | EVD | 3985 | Nyenswah Nagbe | 2014 | Ebola epidemic--Liberia, March-October 2014 | MMWR. Morbidity and mortality weekly report | 1082-1086 | 46 | 63 |  |
| Cases/CFR | RVF | 4017 | Madani | 2003 | Rift Valley fever epidemic in Saudi Arabia: epidemiological, clinical, and laboratory characteristics | Clinical infectious diseases : an official publication of the Infectious Diseases Society of America | 1084-1092 | 8 | 37 | 10.1086/378747 |
| Cases/CFR | EVD | 4065 | Team, W H O Ebola Response | 2016 | Ebola Virus Disease among Male and Female Persons in West Africa | The New England journal of medicine | 96-98 | 1 | 374 | 10.1056/NEJMc1510305 |
| Cases/CFR | HPS | 4139 | Bayard | 2004 | Outbreak of hantavirus pulmonary syndrome, Los Santos, Panama, 1999-2000 | Emerging infectious diseases | 1635-1642 | 9 | 10 | 10.3201/eid1009.040143 |
| Cases/CFR | CCHF | 4153 | Melnik | 2016 | Crimean-Congo Hemorrhagic fever in former Soviet Union countries based on ProMED-RUS reports (2005-2015 years) | International Journal of Infectious Diseases | 183-183 |  | 45 | 10.1016/j.ijid.2016.02.427 |
| Cases/CFR | HPS | 4164 | Pinto Jr. | 2014 | Regional variations and time trends of hantavirus pulmonary syndrome in Brazil | Epidemiology and Infection | 2166-2171 | 10 | 142 | 10.1017/S0950268813003403 |
| Cases/CFR | HPS | 4171 | Martinez | 2010 | Hantavirus pulmonary syndrome in Argentina, 1995-2008 | Emerging infectious diseases | 1853-1860 | 12 | 16 | 10.3201/eid1612.091170 |
| Treatment | EVD | 4180 | van Griensven | 2016 | Evaluation of convalescent plasma for Ebola virus disease in Guinea | The New England journal of medicine | 33-42 | 1 | 374 | 10.1056/NEJMoa1511812 |
| Treatment | HPS | 4274 | Wernly | 2011 | Extracorporeal membrane oxygenation support improves survival of patients with Hantavirus cardiopulmonary syndrome refractory to medical treatment | European journal of cardio-thoracic surgery : official journal of the European Association for Cardio-thoracic Surgery | 1334-1340 | 6 | 40 | 10.1016/j.ejcts.2011.01.089 |
| Cases/CFR | EVD | 4300 | Wong | 2016 | Assessment of the severity of Ebola virus disease in Sierra Leone in 2014-2015 | Epidemiology and infection | 1473-1481 | 7 | 144 | 10.1017/S0950268815003003 |
| Cases/CFR | CCHF | 4374 | Yesilyurt | 2011 | The early prediction of fatality in Crimean Congo hemorrhagic fever patients | Saudi medical journal | 742-743 | 7 | 32 |  |
| Cases/CFR | HFRS | 4391 | Gledovic | 2008 | Hemorrhagic fever with renal syndrome in Montenegro | Japanese journal of infectious diseases | 386-387 | 5 | 61 |  |
| Cases/CFR | HFRS | 4432 | Zhang | 2010 | Hantaviruses in small mammals and humans in the coastal region of Zhejiang Province, China | Journal of medical virology | 987-995 | 6 | 82 | 10.1002/jmv.21737 |
| Cases/CFR | HFRS | 4433 | Zhang | 2010 | Hantavirus infections in humans and animals, China | Emerging infectious diseases | 1195-1203 | 8 | 16 | 10.3201/eid1608.090470 |
| Cases/CFR | HFRS | 4436 | Zheng | 2019 | The characteristics of current natural foci of hemorrhagic fever with renal syndrome in Shandong Province, China, 2012-2015 | PLoS neglected tropical diseases | e0007148-e0007148 | 5 | 13 | 10.1371/journal.pntd.0007148 |
| Cases/CFR | RVF | HS1 | Hassan | 2011 | The 2007 Rift Valley Fever Outbreak in Sudan | PLoS Negl Trop Dis | e1229 | 9 | 5 |  |
| Cases/CFR | LF | HS3 | Bausch | 2001 | Lassa Fever in Guinea: I. Epidemiology of Human Disease and Clinical Observations | Vector Borne and Zoonotic Diseases | 269-281 | 4 | 1 |  |
| Cases/CFR | HFRS | HS4 | Ye | 2021 | Improving the precision of modeling the incidence of hemorrhagic fever with renal syndrome in mainland China with an ensemble machine learning approach | PloS one | e0248597 | 3 | 16 |  |
| Cases/CFR | HFRS | HS5 | Heyman | 2007 | Haemorrhagic fever with renal syndrome: an analysis of the outbreaks in Belgium, France, Germany, the Netherlands and Luxembourg in 2005 | Eurosurveillance | 15-16 | 5 | 12 |  |
| Cases/CFR | ArHF | HS6 | Argentinian website | 2019 | Fiebre hemorrágica argentina | https://save.org.ar/wp-content/uploads/2019/07/Documento-Posicion-Fiebre-Hemorragica-Argentina-.pdf |  |  |  |  |
| Cases/CFR | OHF | HS7 | Růžek | 2010 | Omsk haemorrhagic fever | The Lancet |  | 9758 | 376 |  |
| Cases/CFR | VeHF | HS8 | Venezuelan website | 2012 | Fièvre hémorragique vénézuélienne | https://botica.xyz/24/ |  |  |  |  |
| Cases/CFR | SHF | HS9 | Ellwanger | 2017 | Keeping track of hidden dangers - The short history of the Sabiá virus | Revista da Sociedade Brasileira de Medicina Tropical | 03-août |  | 50 |  |

*Note: AHF, Alkhurma hemorrhagic fever; ArHF, Argentine hemorrhagic fever; BHF, Bolivian hemorrhagic fever; CHF, Chapare hemorrhagic fever; CCHF, Crimean-Congo hemorrhagic fever; EVD, Ebola Virus Disease; HPS, Hantavirus Pulmonary Syndrome; HFRS, Hemorrhagic fever with renal syndrome; LF, Lassa fever; LHF, Lujo hemorrhagic fever; MVD, Marburg virus disease; OHF, Omsk hemorrhagic fever; RVF, Rift Valley fever; SHF, Sabia hemorrhagic fever; VeHF, Venezuelan hemorrhagic fever.*
