# Supplementary material for: The number of cases, mortality and treatments of viral hemorrhagic fevers: A systematic review
Source: PLoS Negl Trop Dis. 2022 Oct 31;16(10):e0010889. doi: 10.1371/journal.pntd.0010889 (PMC9648854; doi:10.1371/journal.pntd.0010889)
Supplement: S2 Table — (DOCX) [file pntd.0010889.s003.docx]

S2 Table. References details of each included WHO or CDC report.

| **VHF** | **Identifiant** | **WHO/CDC** | **Reference type** | **Reference** | **Reference year** | **Secondary reference** |
| --- | --- | --- | --- | --- | --- | --- |
| AHF | W2 [1] | CDC | Report | https://wwwnc.cdc.gov/eid/article/11/5/04-1298_article | 2005 | WHO report - CCHF, hantavirus and Alkhurma - April 2010 |
| BHF | W1 [2] | CDC | Report | https://www.cdc.gov/mmwr/preview/mmwrhtml/00035133.htm | 1994 |  |
| CCHF | W1 [3] | WHO | Website | https://www.who.int/csr/don/2001_06_29e/en/ | 2001 |  |
| CCHF | W2 [4] | WHO | Website | https://www.who.int/csr/don/2003_03_24/en/ | 2003 |  |
| CCHF | W3 [5] | WHO | Website | https://www.who.int/csr/don/2006_08_08b/en/ | 2006 |  |
| CCHF | W4 [6] | WHO | Website | https://www.who.int/csr/don/2010_10_25a/en/ | 2010 |  |
| CHF | W1 [7] | CDC | Website | https://www.cdc.gov/vhf/chapare/index.html | 2019 |  |
| CHF | W2 [7] | CDC | Website | https://www.cdc.gov/vhf/chapare/index.html | 2019 |  |
| EVD | W1 [8] | CDC | Excel file | CDC - West Africa 2014-2016 - CASES-DEATHS | 2016 |  |
| EVD | W2 [8] | CDC | Excel file | CDC - West Africa 2014-2016 - CASES-DEATHS | 2016 |  |
| EVD | W3 [8] | CDC | Excel file | CDC - West Africa 2014-2016 - CASES-DEATHS | 2016 |  |
| EVD | W4 [9] | CDC | Website | https://www.cdc.gov/vhf/ebola/history/chronology.html | 1977 |  |
| EVD | W5 [9] | CDC | Website | https://www.cdc.gov/vhf/ebola/history/chronology.html | 1978 | https://www.who.int/news-room/fact-sheets/detail/ebola-virus-disease |
| EVD | W6 [9] | CDC | Website | https://www.cdc.gov/vhf/ebola/history/chronology.html | 1980 | https://www.who.int/news-room/fact-sheets/detail/ebola-virus-disease |
| EVD | W7 [9] | CDC | Website | https://www.cdc.gov/vhf/ebola/history/chronology.html | 1983 | https://www.who.int/news-room/fact-sheets/detail/ebola-virus-disease |
| EVD | W8 [9] | CDC | Website | https://www.cdc.gov/vhf/ebola/history/chronology.html | 1991 |  |
| EVD | W9 [9] | CDC | Website | https://www.cdc.gov/vhf/ebola/history/chronology.html | 1990 |  |
| EVD | W10 [9] | CDC | Website | https://www.cdc.gov/vhf/ebola/history/chronology.html | 1995 | https://www.who.int/news-room/fact-sheets/detail/ebola-virus-disease |
| EVD | W11 [9] | CDC | Website | https://www.cdc.gov/vhf/ebola/history/chronology.html | 1999 | https://www.who.int/news-room/fact-sheets/detail/ebola-virus-disease |
| EVD | W12 [9] | CDC | Website | https://www.cdc.gov/vhf/ebola/history/chronology.html | 1999 |  |
| EVD | W13 [9] | CDC | Website | https://www.cdc.gov/vhf/ebola/history/chronology.html | 2006 |  |
| EVD | W14 [10] | WHO | Website | https://www.who.int/news-room/fact-sheets/detail/ebola-virus-disease | 1996 | https://www.who.int/docstore/wer/pdf/1996/wer7147.pdf |
| EVD | W15 [9] | CDC | Website | https://www.cdc.gov/vhf/ebola/history/chronology.html | 1999 | https://www.who.int/news-room/fact-sheets/detail/ebola-virus-disease |
| EVD | W16 [9] | CDC | Website | https://www.cdc.gov/vhf/ebola/history/chronology.html | 1999 | https://www.who.int/news-room/fact-sheets/detail/ebola-virus-disease |
| EVD | W17 [9] | CDC | Website | https://www.cdc.gov/vhf/ebola/history/chronology.html | 2002 | WHO report - Uganda February 2001 |
| EVD | W18 [9] | CDC | Website | https://www.cdc.gov/vhf/ebola/history/chronology.html | 2003 | https://www.who.int/news-room/fact-sheets/detail/ebola-virus-disease |
| EVD | W19 [9] | CDC | Website | https://www.cdc.gov/vhf/ebola/history/chronology.html | 2003 | https://www.who.int/news-room/fact-sheets/detail/ebola-virus-disease |
| EVD | W20 [9] | CDC | Website | https://www.cdc.gov/vhf/ebola/history/chronology.html | 2004 | https://www.who.int/news-room/fact-sheets/detail/ebola-virus-disease |
| EVD | W21 [9] | CDC | Website | https://www.cdc.gov/vhf/ebola/history/chronology.html | 2003 | https://www.who.int/news-room/fact-sheets/detail/ebola-virus-disease |
| EVD | W22 [9] | CDC | Website | https://www.cdc.gov/vhf/ebola/history/chronology.html | 2005 |  |
| EVD | W23 [9] | CDC | Website | https://www.cdc.gov/vhf/ebola/history/chronology.html | 2005 | https://www.who.int/news-room/fact-sheets/detail/ebola-virus-disease |
| EVD | W24 [9] | CDC | Website | https://www.cdc.gov/vhf/ebola/history/chronology.html | 2005 | https://www.who.int/news-room/fact-sheets/detail/ebola-virus-disease |
| EVD | W25 [9] | CDC | Website | https://www.cdc.gov/vhf/ebola/history/chronology.html | 2011 | https://www.who.int/news-room/fact-sheets/detail/ebola-virus-disease |
| EVD | W26 [9] | CDC | Website | https://www.cdc.gov/vhf/ebola/history/chronology.html | 2007 | https://www.who.int/news-room/fact-sheets/detail/ebola-virus-disease |
| EVD | W27 [9] | CDC | Website | https://www.cdc.gov/vhf/ebola/history/chronology.html | 2009 | https://www.who.int/news-room/fact-sheets/detail/ebola-virus-disease |
| EVD | W28 [9] | CDC | Website | https://www.cdc.gov/vhf/ebola/history/chronology.html | 2009 |  |
| EVD | W29 [9] | CDC | Website | https://www.cdc.gov/vhf/ebola/history/chronology.html | 2012 |  |
| EVD | W30 [9] | CDC | Website | https://www.cdc.gov/vhf/ebola/history/chronology.html | 2013 | https://www.who.int/news-room/fact-sheets/detail/ebola-virus-disease |
| EVD | W31 [9] | CDC | Website | https://www.cdc.gov/vhf/ebola/history/chronology.html | 2012 | https://www.who.int/news-room/fact-sheets/detail/ebola-virus-disease |
| EVD | W32 [9] | CDC | Website | https://www.cdc.gov/vhf/ebola/history/chronology.html | 2013 | https://www.who.int/news-room/fact-sheets/detail/ebola-virus-disease |
| EVD | W33 [9] | CDC | Website | https://www.cdc.gov/vhf/ebola/history/chronology.html | 2014 |  |
| EVD | W34 [10] | WHO | Website | https://www.who.int/news-room/fact-sheets/detail/ebola-virus-disease | 2019 | https://www.cdc.gov/vhf/ebola/history/chronology.html |
| EVD | W35 [10] | WHO | Website | https://www.who.int/news-room/fact-sheets/detail/ebola-virus-disease | 2019 | https://www.cdc.gov/vhf/ebola/history/chronology.html |
| EVD | W36 [10] | WHO | Website | https://www.who.int/news-room/fact-sheets/detail/ebola-virus-disease | 2019 | https://www.cdc.gov/vhf/ebola/history/chronology.html |
| EVD | W37 [9] | CDC | Website | https://www.cdc.gov/vhf/ebola/history/chronology.html | 2015 | https://www.who.int/news-room/fact-sheets/detail/ebola-virus-disease |
| EVD | W38 [9] | CDC | Website | https://www.cdc.gov/vhf/ebola/history/chronology.html | 2016 | https://www.who.int/news-room/fact-sheets/detail/ebola-virus-disease |
| EVD | W39 [9] | CDC | Website | https://www.cdc.gov/vhf/ebola/history/chronology.html | 2016 | https://www.who.int/news-room/fact-sheets/detail/ebola-virus-disease |
| EVD | W40 [9] | CDC | Website | https://www.cdc.gov/vhf/ebola/history/chronology.html | 2016 | https://www.who.int/news-room/fact-sheets/detail/ebola-virus-disease |
| EVD | W41 [9] | CDC | Website | https://www.cdc.gov/vhf/ebola/history/chronology.html | 2014 | https://www.who.int/news-room/fact-sheets/detail/ebola-virus-disease |
| EVD | W42 [9] | CDC | Website | https://www.cdc.gov/vhf/ebola/history/chronology.html | 2014 | https://www.who.int/news-room/fact-sheets/detail/ebola-virus-disease |
| EVD | W43 [9] | CDC | Website | https://www.cdc.gov/vhf/ebola/history/chronology.html | 2016 | https://www.who.int/news-room/fact-sheets/detail/ebola-virus-disease |
| EVD | W44 [9] | CDC | Website | https://www.cdc.gov/vhf/ebola/history/chronology.html | 2017 | https://www.who.int/news-room/fact-sheets/detail/ebola-virus-disease |
| EVD | W45 [9] | CDC | Website | https://www.cdc.gov/vhf/ebola/history/chronology.html | 2018 | https://www.who.int/news-room/fact-sheets/detail/ebola-virus-disease |
| EVD | W46 [10] | WHO | Report | https://www.who.int/news-room/fact-sheets/detail/ebola-virus-disease | 2019 |  |
| EVD | W47 [10] | WHO | Website | https://www.who.int/news-room/fact-sheets/detail/ebola-virus-disease | 2019 |  |
| HPS | W1 [11] | CDC | Website | https://www.cdc.gov/hantavirus/surveillance/index.html | 2019 |  |
| HPS | W2 [11] | CDC | Website | https://www.cdc.gov/hantavirus/surveillance/index.html | 2019 |  |
| HPS | W3 [11] | CDC | Website | https://www.cdc.gov/hantavirus/surveillance/index.html | 2019 |  |
| HPS | W4 [11] | CDC | Website | https://www.cdc.gov/hantavirus/surveillance/index.html | 2019 |  |
| HPS | W5 [11] | CDC | Website | https://www.cdc.gov/hantavirus/surveillance/index.html | 2019 |  |
| HPS | W6 [11] | CDC | Website | https://www.cdc.gov/hantavirus/surveillance/index.html | 2019 |  |
| HPS | W7 [11] | CDC | Website | https://www.cdc.gov/hantavirus/surveillance/index.html | 2019 |  |
| HPS | W8 [11] | CDC | Website | https://www.cdc.gov/hantavirus/surveillance/index.html | 2019 |  |
| HPS | W9 [11] | CDC | Website | https://www.cdc.gov/hantavirus/surveillance/index.html | 2019 |  |
| HPS | W10 [11] | CDC | Website | https://www.cdc.gov/hantavirus/surveillance/index.html | 2019 |  |
| HPS | W11 [11] | CDC | Website | https://www.cdc.gov/hantavirus/surveillance/index.html | 2019 |  |
| HPS | W12 [11] | CDC | Website | https://www.cdc.gov/hantavirus/surveillance/index.html | 2019 |  |
| HPS | W13 [11] | CDC | Website | https://www.cdc.gov/hantavirus/surveillance/index.html | 2019 |  |
| HPS | W14 [11] | CDC | Website | https://www.cdc.gov/hantavirus/surveillance/index.html | 2019 |  |
| HPS | W15 [11] | CDC | Website | https://www.cdc.gov/hantavirus/surveillance/index.html | 2019 |  |
| HPS | W16 [11] | CDC | Website | https://www.cdc.gov/hantavirus/surveillance/index.html | 2019 |  |
| HPS | W17 [11] | CDC | Website | https://www.cdc.gov/hantavirus/surveillance/index.html | 2019 |  |
| HPS | W18 [11] | CDC | Website | https://www.cdc.gov/hantavirus/surveillance/index.html | 2019 |  |
| HPS | W19 [11] | CDC | Website | https://www.cdc.gov/hantavirus/surveillance/index.html | 2019 |  |
| HPS | W20 [11] | CDC | Website | https://www.cdc.gov/hantavirus/surveillance/index.html | 2019 |  |
| HPS | W21 [11] | CDC | Website | https://www.cdc.gov/hantavirus/surveillance/index.html | 2019 |  |
| HPS | W22 [11] | CDC | Website | https://www.cdc.gov/hantavirus/surveillance/index.html | 2019 |  |
| HPS | W23 [11] | CDC | Website | https://www.cdc.gov/hantavirus/surveillance/index.html | 2019 |  |
| HPS | W24 [11] | CDC | Website | https://www.cdc.gov/hantavirus/surveillance/index.html | 2019 |  |
| HPS | W25 [11] | CDC | Website | https://www.cdc.gov/hantavirus/surveillance/index.html | 2019 |  |
| HPS | W26 [11] | CDC | Website | https://www.cdc.gov/hantavirus/surveillance/index.html | 2019 |  |
| LF | W1 [12] | WHO | Website | https://www.who.int/csr/don/2000_01_18a/en/ | 2000 |  |
| LF | W2 [13] | WHO | Website | https://www.who.int/csr/don/2000_04_04/en/ | 2000 |  |
| LF | W3 [14] | WHO | Website | https://www.who.int/csr/don/2000_03_14/en/ | 2000 |  |
| LF | W4 [15] | WHO | Website | https://www.who.int/csr/don/2000_07_26/en/ | 2000 |  |
| LF | W5 [16] | WHO | Website | https://www.who.int/csr/don/2003_02_10a/en/ | 2003 |  |
| LF | W6 [17] | WHO | Website | https://www.who.int/csr/don/2006_07_25/en/ | 2006 |  |
| LF | W7 [18] | WHO | Website | https://www.who.int/csr/don/2012_04_04/en/ | 2012 |  |
| LF | W8 [19] | WHO | Website | https://www.who.int/csr/don/28-may-2015-lassa-fever-usa/en/ | 2015 |  |
| LF | W9 [20] | WHO | Website | https://www.who.int/csr/don/27-may-2016-lassa-fever-nigeria/en/ | 2016 |  |
| LF | W10 [21] | WHO | Website | https://www.who.int/emergencies/disease-outbreak-news/item/19-february-2016-lassa-fever-benin-en | 2016 |  |
| LF | W11 [22] | WHO | Website | https://www.who.int/csr/don/23-march-2016-lassa-fever-togo/en/ | 2016 |  |
| LF | W12 [23] | WHO | Website | https://www.who.int/csr/don/23-march-2016-lassa-fever-germany/en/ | 2016 |  |
| LF | W13 [24] | WHO | Website | https://www.who.int/csr/don/8-april-2016-lassa-fever-sweden/en/ | 2016 |  |
| LF | W14 [25] | WHO | Website | https://www.who.int/csr/don/22-february-2018-lassa-fever-liberia/en/ | 2018 |  |
| LF | W15 [26] | WHO | Website | https://www.who.int/emergencies/disease-outbreak-news/item/13-june-2016-lassa-fever-benin-en | 2016 |  |
| LF | W16 [27] | WHO | Website | https://www.who.int/csr/don/10-march-2017-lassa-fever-benin-togo-burkina-faso/en/ | 2017 |  |
| LF | W17 [27] | WHO | Website | https://www.who.int/csr/don/10-march-2017-lassa-fever-benin-togo-burkina-faso/en/ | 2017 |  |
| LF | W18 [28] | WHO | Website | https://www.who.int/csr/don/28-june-2017-lassa-fever-nigeria/en/ | 2017 |  |
| LF | W19 [29] | WHO | Website | https://www.who.int/csr/don/22-february-2018-lassa-fever-liberia/en/ | 2018 |  |
| LF | W20 [30] | WHO | Website | https://www.who.int/csr/don/20-april-2018-lassa-fever-nigeria/en/ | 2018 |  |
| LF | W21 [31] | WHO | Website | https://www.who.int/csr/don/14-february-2019-lassa-fever-nigeria/en/ | 2019 |  |
| LF | W22 [32] | WHO | Website | https://www.who.int/emergencies/disease-outbreak-news/item/1996_07_30a-en | 1996 |  |
| LF | W23 [33] | WHO | Website | https://www.who.int/emergencies/disease-outbreak-news/item/1997_05_22-en | 1997 |  |
| LHF | W1 [34] | CDC | Website | https://www.cdc.gov/vhf/lujo/index.html | 2013 |  |
| MVD | W1 [35] | CDC | Website | https://www.cdc.gov/vhf/marburg/outbreaks/chronology.html | 2019 | https://www.who.int/en/news-room/fact-sheets/detail/marburg-virus-disease |
| MVD | W2 [35] | CDC | Website | https://www.cdc.gov/vhf/marburg/outbreaks/chronology.html | 2019 | https://www.who.int/en/news-room/fact-sheets/detail/marburg-virus-disease |
| MVD | W3 [35] | CDC | Website | https://www.cdc.gov/vhf/marburg/outbreaks/chronology.html | 2019 | https://www.who.int/en/news-room/fact-sheets/detail/marburg-virus-disease |
| MVD | W4 [35] | CDC | Website | https://www.cdc.gov/vhf/marburg/outbreaks/chronology.html | 2019 | https://www.who.int/en/news-room/fact-sheets/detail/marburg-virus-disease |
| MVD | W5 [35] | CDC | Website | https://www.cdc.gov/vhf/marburg/outbreaks/chronology.html | 2019 |  |
| MVD | W6 [35] | CDC | Website | https://www.cdc.gov/vhf/marburg/outbreaks/chronology.html | 2019 | https://www.who.int/en/news-room/fact-sheets/detail/marburg-virus-disease |
| MVD | W7 [35] | CDC | Website | https://www.cdc.gov/vhf/marburg/outbreaks/chronology.html | 2019 | https://www.who.int/en/news-room/fact-sheets/detail/marburg-virus-disease |
| MVD | W8 [35] | CDC | Website | https://www.cdc.gov/vhf/marburg/outbreaks/chronology.html | 2019 | https://www.who.int/en/news-room/fact-sheets/detail/marburg-virus-disease |
| MVD | W9 [35] | CDC | Website | https://www.cdc.gov/vhf/marburg/outbreaks/chronology.html | 2019 | https://www.who.int/en/news-room/fact-sheets/detail/marburg-virus-disease |
| MVD | W10 [35] | CDC | Website | https://www.cdc.gov/vhf/marburg/outbreaks/chronology.html | 2019 | https://www.who.int/en/news-room/fact-sheets/detail/marburg-virus-disease |
| MVD | W11 [35] | CDC | Website | https://www.cdc.gov/vhf/marburg/outbreaks/chronology.html | 2019 | https://www.who.int/en/news-room/fact-sheets/detail/marburg-virus-disease |
| MVD | W12 [35] | CDC | Website | https://www.cdc.gov/vhf/marburg/outbreaks/chronology.html | 2019 | https://www.who.int/en/news-room/fact-sheets/detail/marburg-virus-disease |
| MVD | W13 [36] | CDC | Website | https://www.cdc.gov/ncezid/dhcpp/vspb/outbreaks.html | 2019 |  |
| RVF | W1 [37] | CDC | Website | https://www.cdc.gov/mmwr/preview/mmwrhtml/00051976.htm | 1998 |  |
| RVF | W2 [37] | CDC | Website | https://www.cdc.gov/mmwr/preview/mmwrhtml/00051976.htm | 1998 |  |
| RVF | W3 [37] | CDC | Website | https://www.cdc.gov/mmwr/preview/mmwrhtml/00051976.htm | 1998 |  |
| RVF | W4 [38] | CDC | Website | https://www.cdc.gov/mmwr/preview/mmwrhtml/mm4943a3.htm | 2000 | https://www.who.int/en/news-room/fact-sheets/detail/rift-valley-fever |
| RVF | W5 [39] | CDC | Website | https://www.cdc.gov/vhf/rvf/outbreaks/summaries.html | 2019 |  |
| RVF | W6 [39] | CDC | Website | https://www.cdc.gov/vhf/rvf/outbreaks/summaries.html | 2019 |  |
| RVF | W7 [39] | CDC | Website | https://www.cdc.gov/vhf/rvf/outbreaks/summaries.html | 2019 | https://www.who.int/en/news-room/fact-sheets/detail/rift-valley-fever |
| RVF | W8 [39] | CDC | Website | https://www.cdc.gov/vhf/rvf/outbreaks/summaries.html | 2019 | https://www.who.int/en/news-room/fact-sheets/detail/rift-valley-fever |
| RVF | W9 [39] | CDC | Website | https://www.cdc.gov/vhf/rvf/outbreaks/summaries.html | 2019 |  |
| RVF | W10 [39] | CDC | Website | https://www.cdc.gov/vhf/rvf/outbreaks/summaries.html | 2019 |  |
| RVF | W11 [39] | CDC | Website | https://www.cdc.gov/vhf/rvf/outbreaks/summaries.html | 2019 | https://www.who.int/en/news-room/fact-sheets/detail/rift-valley-fever |
| RVF | W12 [40] | WHO | Website | https://www.who.int/en/news-room/fact-sheets/detail/rift-valley-fever | 2018 |  |
| RVF | W13 [40] | WHO | Website | https://www.who.int/en/news-room/fact-sheets/detail/rift-valley-fever | 2018 |  |
| RVF | W14 [40] | WHO | Website | https://www.who.int/en/news-room/fact-sheets/detail/rift-valley-fever | 2018 |  |
| RVF | W15 [40] | WHO | Website | https://www.who.int/en/news-room/fact-sheets/detail/rift-valley-fever | 2018 |  |
| RVF | W16 [40] | WHO | Website | https://www.who.int/en/news-room/fact-sheets/detail/rift-valley-fever | 2018 |  |
| RVF | W17 [40] | WHO | Website | https://www.who.int/en/news-room/fact-sheets/detail/rift-valley-fever | 2008 |  |
| RVF | W18 [40] | WHO | Website | https://www.who.int/en/news-room/fact-sheets/detail/rift-valley-fever | 2008 |  |
| RVF | W19 [40] | WHO | Website | https://www.who.int/en/news-room/fact-sheets/detail/rift-valley-fever | 2018 |  |
| RVF | W20 [41] | WHO | Website | https://www.who.int/csr/don/02-august-2016-rift-valley-fever-china/en/ | 2016 |  |
| RVF | W21 [42] | WHO | Website | https://www.who.int/csr/don/26-february-2018-rift-valley-fever-gambia/en/ | 2018 |  |
| RVF | W22 [43] | WHO | Website | https://www.who.int/emergencies/disease-outbreak-news/item/18-june-2018-rift-valley-fever-kenya-en | 2018 |  |
| RVF | W23 [44] | WHO | Website | https://www.who.int/csr/don/13-may-2019-rift-valley-fever-mayotte-france/en/ | 2019 |  |

*Note: AHF, Alkhurma hemorrhagic fever; ArHF, Argentine hemorrhagic fever; BHF, Bolivian hemorrhagic fever; CHF, Chapare hemorrhagic fever; CCHF, Crimean-Congo hemorrhagic fever; EVD, Ebola Virus Disease; HPS, Hantavirus Pulmonary Syndrome; HFRS, Hemorrhagic fever with renal syndrome; LF, Lassa fever; LHF, Lujo hemorrhagic fever; MVD, Marburg virus disease; OHF, Omsk hemorrhagic fever; RVF, Rift Valley fever; SHF, Sabia hemorrhagic fever; VeHF, Venezuelan hemorrhagic fever.*

References

1. Charrel RN, Zaki AM, Fakeeh M, Yousef AI, Chesse R de, Attoui H, et al. Low Diversity of Alkhurma Hemorrhagic Fever Virus, Saudi Arabia, 1994–1999 - Volume 11, Number 5—May 2005 - Emerging Infectious Diseases journal - CDC. [cited 2022 Oct 24]; Available from: https://wwwnc.cdc.gov/eid/article/11/5/04-1298_article

2. CDC. International Notes Bolivian Hemorrhagic Fever -- El Beni Department, Bolivia, 1994 [Internet]. [cited 2022 Oct 24]. Available from: https://www.cdc.gov/mmwr/preview/mmwrhtml/00035133.htm

3. WHO. Disease Outbreak News - Crimean-Congo haemorrhagic fever in Kosovo [Internet]. 2001 [cited 2019 Dec 1]. Available from: https://www.who.int/csr/don/2001_06_29e/en/

4. WHO. Disease Outbreak News - Crimean-Congo haemorrhagic fever in Mauritania [Internet]. 2003 [cited 2019 Dec 1]. Available from: https://www.who.int/csr/don/2003_03_24/en/

5. WHO. Disease Outbreak News - Crimean-Congo haemorrhagic fever in Turkey [Internet]. 2006 [cited 2019 Dec 1]. Available from: https://www.who.int/csr/don/2006_08_08b/en/

6. WHO. Disease Outbreak News - Crimean-Congo haemorrhagic fever in Pakistan [Internet]. 2010 [cited 2019 Dec 1]. Available from: https://www.who.int/csr/don/2010_10_25a/en/

7. CDC. Chapare Hemorrhagic Fever (CHHF) [Internet]. 2019 Nov [cited 2019 Dec 1]. Available from: https://www.cdc.gov/vhf/chapare/index.html

8. WHO. Ebola measure, Cases, Deaths - Guinea, Liberia, Sierra Leone - 2014-2016 outbreak [Internet]. [cited 2019 Nov 29]. Available from: https://apps.who.int/gho/athena/xmart/EBOLA_MEASURE/CASES,DEATHS?filter=COUNTRY:GIN;COUNTRY:UNSPECIFIED;COUNTRY:LBR;COUNTRY:UNSPECIFIED;COUNTRY:SLE;COUNTRY:UNSPECIFIED;LOCATION:-;DATAPACKAGEID:2016-05-11;INDICATOR_TYPE:SITREP_CUMULATIVE;INDICATOR_TYPE:SITREP_CUMULATIVE_21_DAYS;SEX:-&format=xml&profile=excel

9. CDC. History of Ebola Virus Disease (EVD) Outbreaks [Internet]. [cited 2019 Dec 1]. Available from: https://www.cdc.gov/vhf/ebola/history/chronology.html

10. WHO. Ebola virus disease - Table: Chronology of previous Ebola virus disease outbreaks [Internet]. [cited 2019 Dec 1]. Available from: https://www.who.int/news-room/fact-sheets/detail/ebola-virus-disease

11. CDC. Reported Cases of Hantavirus Disease [Internet]. [cited 2019 Dec 1]. Available from: https://www.cdc.gov/hantavirus/surveillance/index.html

12. WHO. Disease Outbreak News - Lassa fever in Germany ex-Africa (Côte d’Ivoire and Ghana) [Internet]. 2016 [cited 2019 Dec 1]. Available from: https://www.who.int/emergencies/disease-outbreak-news/item/19-february-2016-lassa-fever-benin-en

13. WHO. Disease Outbreak News - Lassa fever in Germany ex-Nigeria [Internet]. 2000 [cited 2019 Dec 1]. Available from: https://www.who.int/csr/don/2000_04_04/en/

14. WHO. Disease Outbreak News - Lassa fever in UK ex-Sierra Leone [Internet]. 2000 [cited 2019 Dec 1]. Available from: https://www.who.int/csr/don/2000_03_14/en/

15. WHO. Disease Outbreak News - Lassa fever in Netherlands ex-Sierra Leone [Internet]. 2000 [cited 2019 Dec 1]. Available from: https://www.who.int/csr/don/2000_07_26/en/

16. WHO. Disease Outbreak News - Lassa fever in UK ex-Sierra Leone [Internet]. 2003 [cited 2019 Dec 1]. Available from: https://www.who.int/csr/don/2003_02_10a/en/

17. WHO. Disease Outbreak News - Lassa fever in Germany ex-Sierra Leone [Internet]. 2006 [cited 2019 Dec 1]. Available from: https://www.who.int/csr/don/2006_07_25/en/

18. WHO. Disease Outbreak News - Lassa fever in Nigeria [Internet]. 2012 [cited 2019 Dec 1]. Available from: https://www.who.int/csr/don/2012_04_04/en/

19. WHO. Disease Outbreak News - Lassa fever in USA ex-Liberia [Internet]. 2015 [cited 2019 Dec 1]. Available from: https://www.who.int/csr/don/2012_04_04/en/

20. WHO. Disease Outbreak News - Lassa fever in Nigeria [Internet]. 2016 [cited 2019 Dec 1]. Available from: https://www.who.int/csr/don/27-may-2016-lassa-fever-nigeria/en/

21. WHO. Disease Outbreak News - Lassa fever in Benin [Internet]. 2016 [cited 2019 Dec 1]. Available from: https://www.who.int/emergencies/disease-outbreak-news/item/19-february-2016-lassa-fever-benin-en

22. WHO. Disease Outbreak News - Lassa fever in Togo [Internet]. 2016 [cited 2019 Dec 1]. Available from: https://www.who.int/csr/don/23-march-2016-lassa-fever-togo/en/

23. WHO. Disease Outbreak News - Lassa fever in Germany [Internet]. 2016 [cited 2019 Dec 1]. Available from: https://www.who.int/csr/don/23-march-2016-lassa-fever-germany/en/

24. WHO. Disease Outbreak News - Lassa fever in Sweden ex-Liberia [Internet]. 2016 [cited 2019 Dec 1]. Available from: https://www.who.int/csr/don/8-april-2016-lassa-fever-sweden/en/

25. WHO. Disease Outbreak News - Lassa fever in Liberia [Internet]. 2018 [cited 2019 Dec 1]. Available from: https://www.who.int/csr/don/22-february-2018-lassa-fever-liberia/en/

26. WHO. Disease Outbreak News - Lassa fever in Benin [Internet]. 2016 [cited 2019 Dec 1]. Available from: https://www.who.int/emergencies/disease-outbreak-news/item/13-june-2016-lassa-fever-benin-en

27. WHO. Disease Outbreak News - Lassa fever in Benin, Togo, Burkina Faso [Internet]. 2017 [cited 2019 Dec 1]. Available from: https://www.who.int/csr/don/10-march-2017-lassa-fever-benin-togo-burkina-faso/en/

28. WHO. Disease Outbreak News - Lassa fever in Nigeria [Internet]. 2017 [cited 2019 Dec 1]. Available from: https://www.who.int/csr/don/28-june-2017-lassa-fever-nigeria/en/

29. WHO. Disease Outbreak News - Lassa fever in Liberia ex-Guinea [Internet]. 2018 [cited 2019 Dec 1]. Available from: https://www.who.int/csr/don/22-february-2018-lassa-fever-liberia/en/

30. WHO. Disease Outbreak News - Lassa fever in Nigeria [Internet]. 2018 [cited 2019 Dec 1]. Available from: https://www.who.int/csr/don/20-april-2018-lassa-fever-nigeria/en/

31. WHO. Disease Outbreak News - Lassa fever in Nigeria [Internet]. 2019 [cited 2019 Dec 1]. Available from: https://www.who.int/csr/don/14-february-2019-lassa-fever-nigeria/en/

32. WHO. Disease Outbreak News - Lassa fever in Sierra Leone [Internet]. 1996 [cited 2019 Dec 1]. Available from: https://www.who.int/emergencies/disease-outbreak-news/item/1996_07_30a-en

33. WHO. Disease Outbreak News - Lassa fever in Sierra Leone [Internet]. 1997 [cited 2019 Dec 1]. Available from: https://www.who.int/emergencies/disease-outbreak-news/item/1997_05_22-en

34. CDC. Lujo Hemorrhagic Fever (LUHF) [Internet]. 2013 [cited 2019 Dec 1]. Available from: https://www.cdc.gov/vhf/lujo/index.html

35. CDC. History of Marburg Virus Disease (MVD) Outbreaks [Internet]. [cited 2019 Dec 1]. Available from: https://www.cdc.gov/vhf/marburg/outbreaks/chronology.html

36. CDC. Diseases Caused by High-Consequence Viruses [Internet]. [cited 2019 Dec 1]. Available from: https://www.cdc.gov/ncezid/dhcpp/vspb/diseases.html

37. CDC. Rift Valley Fever -- East Africa, 1997-1998 [Internet]. [cited 2019 Dec 1]. Available from: https://www.cdc.gov/mmwr/preview/mmwrhtml/00051976.htm

38. CDC. Update: Outbreak of Rift Valley Fever --- Saudi Arabia, August--November 2000 [Internet]. [cited 2019 Dec 1]. Available from: https://www.cdc.gov/mmwr/preview/mmwrhtml/mm4943a3.htm

39. CDC. Rift Valley Fever (RVF) - Outbreak Summaries [Internet]. [cited 2019 Dec 1]. Available from: https://www.cdc.gov/vhf/rvf/outbreaks/summaries.html

40. WHO. Rift Valley fever - Outbreaks that have occurred since 2000 [Internet]. 2018 [cited 2019 Dec 1]. Available from: https://www.who.int/en/news-room/fact-sheets/detail/rift-valley-fever

41. WHO. Disease Outbreak News - Rift Valley fever in China ex-Angola [Internet]. 2016 [cited 2019 Dec 1]. Available from: https://www.who.int/csr/don/02-august-2016-rift-valley-fever-china/en/

42. WHO. Disease Outbreak News - Rift Valley fever in Gambia [Internet]. 2018 [cited 2019 Dec 1]. Available from: https://www.who.int/csr/don/26-february-2018-rift-valley-fever-gambia/en/

43. WHO. Rift Valley fever – Kenya [Internet]. 2018 [cited 2019 Dec 1]. Available from: https://www.who.int/emergencies/disease-outbreak-news/item/18-june-2018-rift-valley-fever-kenya-en

44. WHO. Disease Outbreak News - Rift Valley fever in Mayotte [Internet]. 2019 [cited 2019 Dec 1]. Available from: https://www.who.int/csr/don/02-august-2016-rift-valley-fever-china/en/
