# Supplementary material for: The number of cases, mortality and treatments of viral hemorrhagic fevers: A systematic review
Source: PLoS Negl Trop Dis. 2022 Oct 31;16(10):e0010889. doi: 10.1371/journal.pntd.0010889 (PMC9648854; doi:10.1371/journal.pntd.0010889)
Supplement: S3 Table — (DOCX) [file pntd.0010889.s004.docx]

S3 Table. Quality Assessment of studies reporting results on treatments – Randomized Trials

| **Reference** | **VHF** | **Randomization process** | **Deviations from intended interventions** | **Missing outcome data** | **Measurement of the outcome** | **Selection of the reported result** | **Overall Bias** |
| --- | --- | --- | --- | --- | --- | --- | --- |
| Davey 2016 [1] | EVD | Low | Low | Low | Low | Low | Low |
| Gui 1987 [2] | HFRS | Some concerns | Low | Low | Low | Some concerns | Some concerns |
| Huggins 1991 [3] | HFRS | Some concerns | Low | Low | Low | Low | Some concerns |
| Koksal 2010 [4] | CCHF | Some concerns | Low | Low | Low | Some concerns | Some concerns |
| Maiztegui 1979 [5] | ArHF | Some concerns | Low | Low | Low | Some concerns | Some concerns |
| McCormick 1986 [6] | LF | Low | Some concerns | Low | Low | Some concerns | Some concerns |
| Mertz 2004 [7] | HPS | Some concerns | Low | Low | Low | Some concerns | Some concerns |
| Mulangu 2019 [8] | EVD | Low | Low | Low | Low | Low | Low |
| Salehi 2013 [9] | CCHF | Some concerns | Some concerns | Low | Some concerns | Some concerns | Some concerns |
| Vial 2013 [10] | HPS | Low | Low | Low | Low | Some concerns | Some concerns |

*Note: ArHF, Argentine hemorrhagic fever; CCHF, Crimean-Congo hemorrhagic fever; EVD, Ebola Virus Disease; HPS, Hantavirus Pulmonary Syndrome; HFRS, Hemorrhagic fever with renal syndrome; LF, Lassa fever.*

References

1. Davey R, Dodd L, Proschan M, Neaton J, Neuhaus Nordwall J, Koopmeiners J, et al. A Randomized, Controlled Trial of ZMapp for Ebola Virus Infection. N Engl J Med. 2016;375(15):1448‐1456.

2. Gui X, Ho M, Cohen M, Wang Q, Huang H, Xie Q. Hemorrhagic fever with renal syndrome: treatment with recombinant alpha interferon. J Infect Dis. 1987;155(5):1047‐1051.

3. Huggins JW, Hsiang CM, Cosgriff TM, Guang MY, Smith JI, Wu ZO, et al. Prospective, double-blind, concurrent, placebo-controlled clinical trial of intravenous ribavirin therapy of hemorrhagic fever with renal syndrome. J Infect Dis. 1991 Dec;164(6):1119–27.

4. Koksal I, Yilmaz G, Aksoy F, Aydin H, Yavuz I, Iskender S, et al. The efficacy of ribavirin in the treatment of Crimean-Congo hemorrhagic fever in Eastern Black Sea region in Turkey. J Clin Virol Off Publ Pan Am Soc Clin Virol. 2010 Jan;47(1):65–8.

5. Maiztegui JI, Fernandez NJ, de Damilano AJ. Efficacy of immune plasma in treatment of Argentine haemorrhagic fever and association between treatment and a late neurological syndrome. Lancet Lond Engl. 1979 Dec 8;2(8154):1216–7.

6. McCormick JB, King IJ, Webb PA, Scribner CL, Craven RB, Johnson KM, et al. Lassa fever. Effective therapy with ribavirin. N Engl J Med. 1986 Jan 2;314(1):20–6.

7. Mertz G, Miedzinski L, Goade D, Pavia A, Hjelle B, Hansbarger C, et al. Placebo-controlled, double-blind trial of intravenous ribavirin for the treatment of hantavirus cardiopulmonary syndrome in North America. Clin Infect Dis. 2004;39(9):1307‐1313.

8. Mulangu S, Dodd LE, Davey RT Jr, Tshiani Mbaya O, Proschan M, Mukadi D, et al. A Randomized, Controlled Trial of Ebola Virus Disease Therapeutics. N Engl J Med. 2019 Dec 12;381(24):2293–303.

9. Salehi H, Salehi MM, Adibi N, Salehi MM, H. S, M. S, et al. Comparative study between Ribavirin and Ribavirin plus Intravenous Immunoglobulin against Crimean Congo hemorrhagic fever. J Res Med Sci. 2013 Jun;18(6):497–500.

10. Vial P, Valdivieso F, Ferres M, Riquelme R, Rioseco M, Calvo M, et al. High-dose intravenous methylprednisolone for hantavirus cardiopulmonary syndrome in Chile: a double-blind, randomized controlled clinical trial. Clin Infect Dis. 2013;57(7):943‐951.
