# Supplementary material for: The number of cases, mortality and treatments of viral hemorrhagic fevers: A systematic review
Source: PLoS Negl Trop Dis. 2022 Oct 31;16(10):e0010889. doi: 10.1371/journal.pntd.0010889 (PMC9648854; doi:10.1371/journal.pntd.0010889)
Supplement: S4 Table — (DOCX) [file pntd.0010889.s005.docx]

S4 Table. Quality Assessment of studies reporting results on treatments – Non-Randomized Trials

| **Study name** | **VHF** | **Selection** | | | | **Comparability** | **Outcome** | | | | **Quality rating** |
| --- | --- | --- | --- | --- | --- | --- | --- | --- | --- | --- | --- |
|  |  | **Representativeness of the exposed cohort** | **Selection of the non-exposed cohort** | **Ascertainment of exposure** | **Demonstration that outcome of interest was not present at start of study** | **Comparability of cohorts on the basis of the design or analysis controlled for confounders** | **Assessment of outcome** | **Was follow-up long enough for outcomes to occur** | **Median duration of follow-up** | **Adequacy of follow-up of cohorts** |  |
| Aluisio 2019 [1] | EVD | D | C | D | B | C | B | B | Not reported | B | Good quality |
| Aluisio 2019 [2] | EVD | B | A | E | B | B | B | B | 28 days | B | Good quality |
| Aluisio 2019 [3] | EVD | B | B | A | B | C | B | B | ETU care | B | Good quality |
| Aluisio 2020 [4] | EVD | B | B | A | B | C | B | B | ETU care | B | Good quality |
| Bai 2016 [5] | EVD | B | C | E | B | B | B | A | 2 months | B | Poor quality |
| Cevik 2008 [6] | CCHF | B | C | E | B | B | B | A | Not reported | B | Poor quality |
| Chapman 1999 [7] | HPS | B | C | E | B | B | B | A | 1 month | B | Poor quality |
| Dokuzoguz 2013 [8] | CCHF | B | A | A | B | C | B | B | Not reported | B | Fair quality |
| Du 2013 [9] | HFRS | B | C | E | B | B | B | A | Not reported | B | Poor quality |
| Dunning 2016 [10] | EVD | C | C | A | B | B | B | A | 14 days | B | Poor quality |
| Dunning 2016 [11] | EVD | B | C | A | B | B | B | A | 7 days | B | Poor quality |
| Elaldi 2009 [12] | CCHF | B | C | E | B | B | B | A | 1 month | B | Fair quality |
| Garbern 2019 [13] | EVD | B | C | E | B | B | B | A | Not reported | B | Fair quality |
| Gignoux 2015 [14] | EVD | B | C | A | B | B | B | A | Not reported | B | Good quality |
| van Griensven 2016 [15] | EVD | B | C | E | B | B | B | A | 1 day | B | Good quality |
| Izadi 2009 [16] | CCHF | B | C | D | B | B | B | A | 10 days | B | Poor quality |
| Kerber 2019 [17] | EVD | B | C | E | B | B | B | A | 10 days | B | Good quality |
| Konde 2017 [18] | EVD | B | C | D | B | B | B | A | Not reported | B | Poor quality |
| Ilori 2019 [19] | LF | B | C | A | B | B | B | A | Not reported | B | Good quality |
| Mardani 2003 [20] | CCHF | B | C | D | B | B | B | A | 10 days | B | Poor quality |
| Sadek 1999 [21] | EVD | B | C | E | B | B | B | A | Not reported | B | Fair quality |
| Sahr 2017 [22] | EVD | B | C | A | B | B | B | A | Not reported | B | Fair quality |
| Sissoko 2016 [23] | EVD | B | C | E | B | B | B | A | On trial | B | Good quality |
| Tulek 2012 [24] | CCHF | B | C | A | B | B | B | A | Minimum 48h | B | Poor quality |
| Vial 2015 [25] | HPS | B | C | E | B | B | B | A | 3 days | B | Poor quality |
| Wernly 2011 [26] | HPS | B | C | A | B | B | B | A | Not reported | B | Poor quality |
| Yam 2020 [27] | EVD | B | C | E | B | B | B | A | ETU care | B | Good quality |
| Yilmaz 2016 [28] | CCHF | B | C | E | B | B | B | A | Not reported | B | Poor quality |

*Note: CCHF, Crimean-Congo hemorrhagic fever; EVD, Ebola Virus Disease; HPS, Hantavirus Pulmonary Syndrome; HFRS, Hemorrhagic fever with renal syndrome; LF, Lassa fever.*

References

1. Aluisio A.R., Yam D., Peters J., Cho D., Perera S., Kennedy S., et al. Association between vitamin a supplementation and mortality in Ebola virus disease: A multisite cohort study. Acad Emerg Med. 2019;26:S67.

2. Aluisio A.R., Yam D., Peters J.L., Cho D.K., Perera S.M., Kennedy S.B., et al. Impact of Intravenous Fluid Therapy on Survival Among Patients with Ebola Virus Disease: An International Multisite Retrospective Cohort Study. Clin Infect Dis Off Publ Infect Dis Soc Am. 2019;ciz344.

3. Aluisio AR, Perera SM, Yam D, Garbern S, Peters JL, Abel L, et al. Vitamin A Supplementation Was Associated with Reduced Mortality in Patients with Ebola Virus Disease during the West African Outbreak. J Nutr. 2019 Oct 1;149(10):1757–65.

4. Aluisio A.R., Perera S.M., Yam D., Garbern S., Peters J.L., Abel L., et al. Association Between Treatment with Oral Third-Generation Cephalosporin Antibiotics and Mortality Outcomes in Ebola Virus Disease: A Multinational Retrospective Cohort Study. Trop Med Int Health TM IH. 2020;10.1111/tmi.13369.

5. Bai C.-Q., Mu J.-S., Kargbo D., Song Y.-B., Niu W.-K., Nie W.-M., et al. Clinical and Virological Characteristics of Ebola Virus Disease Patients Treated with Favipiravir (T-705) - Sierra Leone, 2014. Clin Infect Dis. 2016;63(10):1288–94.

6. Cevik MA, Elaldi N, Akinci E, Ongürü P, Erbay A, Buzgan T, et al. A preliminary study to evaluate the effect of intravenous ribavirin treatment on survival rates in Crimean-Congo hemorrhagic fever. J Infect. 2008 Oct;57(4):350–1.

7. Chapman LE, Mertz GJ, Peters CJ, Jolson HM, Khan AS, Ksiazek TG, et al. Intravenous ribavirin for hantavirus pulmonary syndrome: safety and tolerance during 1 year of open-label experience. Ribavirin Study Group. Antivir Ther. 1999;4(4):211–9.

8. Dokuzoguz B, Celikbas AK, Gök ŞE, Baykam N, Eroglu MN, Ergönül Ö. Severity scoring index for Crimean-Congo hemorrhagic fever and the impact of ribavirin and corticosteroids on fatality. Clin Infect Dis Off Publ Infect Dis Soc Am. 2013 Nov;57(9):1270–4.

9. Du H., Li J., Yu H.-T., Jia Z.-S., Yu D.-H., Wang J.-P., et al. The optimal timing of RRT for critical patients with hemorrhagic fever with renal syndrome. Ther Apher Dial. 2013;17(5):A2.

10. Dunning J, Kennedy SB, Antierens A, Whitehead J, Ciglenecki I, Carson G, et al. Experimental Treatment of Ebola Virus Disease with Brincidofovir. PloS One. 2016 Sep 9;11(9):e0162199–e0162199.

11. Dunning J, Sahr F, Rojek A, Gannon F, Carson G, Idriss B, et al. Experimental Treatment of Ebola Virus Disease with TKM-130803: A Single-Arm Phase 2 Clinical Trial. PLoS Med. 2016 Apr 19;13(4):e1001997–e1001997.

12. Elaldi N, Bodur H, Ascioglu S, Celikbas A, Ozkurt Z, Vahaboglu H, et al. Efficacy of oral ribavirin treatment in Crimean-Congo haemorrhagic fever: a quasi-experimental study from Turkey. J Infect. 2009 Mar;58(3):238–44.

13. Garbern SC, Yam D, Aluisio AR, Cho DK, Kennedy SB, Massaquoi M, et al. Effect of Mass Artesunate-Amodiaquine Distribution on Mortality of Patients With Ebola Virus Disease During West African Outbreak. Open Forum Infect Dis. 2019 May 24;6(7):ofz250–ofz250.

14. Gignoux E., Azman A.S., Ciglenecki I. Artesunate-amodiaquine is associated with reduced ebola mortality. Am J Trop Med Hyg. 2015;93(4):446.

15. van Griensven J, Edwards T, de Lamballerie X, Semple MG, Gallian P, Baize S, et al. Evaluation of Convalescent Plasma for Ebola Virus Disease in Guinea. N Engl J Med. 2016 Jan 7;374(1):33–42.

16. Izadi S, Salehi M, S. I, M. S. Evaluation of the efficacy of ribavirin therapy on survival of Crimean-Congo hemorrhagic fever patients: A case-control study. Jpn J Infect Dis. 2009 Jan;62(1):11–5.

17. Kerber R, Lorenz E, Duraffour S, Sissoko D, Rudolf M, Jaeger A, et al. Laboratory Findings, Compassionate Use of Favipiravir, and Outcome in Patients With Ebola Virus Disease, Guinea, 2015-A Retrospective Observational Study. J Infect Dis. 2019 Jun 19;220(2):195–202.

18. Konde MK, Baker DP, Traore FA, Sow MS, Camara A, Barry AA, et al. Interferon β-1a for the treatment of Ebola virus disease: A historically controlled, single-arm proof-of-concept trial. PloS One. 2017 Feb 22;12(2):e0169255–e0169255.

19. Ilori E.A., Furuse Y., Ipadeola O.B., Dan-Nwafor C.C., Abubakar A., Womi-Eteng O.E., et al. Epidemiologic and clinical features of lassa fever outbreak in Nigeria, january 1-may 6, 2018. Emerg Infect Dis. 2019;25(6):1066–74.

20. Mardani M, Jahromi MK, Naieni KH, Zeinali M. The efficacy of oral ribavirin in the treatment of crimean-congo hemorrhagic fever in Iran. Clin Infect Dis Off Publ Infect Dis Soc Am. 2003 Jun 15;36(12):1613–8.

21. Sadek RF, Khan AS, Stevens G, Peters CJ, Ksiazek TG. Ebola hemorrhagic fever, Democratic Republic of the Congo, 1995: determinants of survival. J Infect Dis. 1999 Feb;179 Suppl 1:S24–7.

22. Sahr F, Ansumana R, Massaquoi TA, Idriss BR, Sesay FR, Lamin JM, et al. Evaluation of convalescent whole blood for treating Ebola Virus Disease in Freetown, Sierra Leone. J Infect. 2017 Mar;74(3):302–9.

23. Sissoko D., Laouenan C., Folkesson E., M’Lebing A.-B., Beavogui A.-H., Baize S., et al. Experimental Treatment with Favipiravir for Ebola Virus Disease (the JIKI Trial): A Historically Controlled, Single-Arm Proof-of-Concept Trial in Guinea. PLoS Med. 2016;13(3).

24. Tulek N., Ozturk B., Bulut C., Tuncer Ertem G., Erdinc F.S., Altun S., et al. The evaluation of ribavirin use in patients with Crimean-Congo haemorrhagic fever. Clin Microbiol Infect. 2012;18:579–80.

25. P.A. V, F. V, M. C, M.L. R, R. R, A. A, et al. A non-randomized multicentre trial of human immune plasma for treatment of hantavirus cardiopulmonary syndrome caused by Andes virus. Antivir Ther. 2015;20(4):377–86.

26. Wernly JA, Dietl CA, Tabe CE, Pett SB, Crandall C, Milligan K, et al. Extracorporeal membrane oxygenation support improves survival of patients with Hantavirus cardiopulmonary syndrome refractory to medical treatment. Eur J Cardio-Thorac Surg Off J Eur Assoc Cardio-Thorac Surg. 2011 Dec;40(6):1334–40.

27. Yam D., Aluisio A.R., Perera S.M., Peters J.L., Cho D.K., Kennedy S.B., et al. Association between multivitamin supplementation and mortality among patients with Ebola virus disease: An international multisite cohort study. Afr J Emerg Med. 2020;23–9.

28. Yilmaz G., Sunbul M., Yapar D., Baykam N., Hasanoglu I., Guner R., et al. Ribavirin in treatment of crimean-congo hemorrhagic fever (CCHF): An international multicenter retrospective analysis. Open Forum Infect Dis. 2016;3.
