# Supplementary material for: The number of cases, mortality and treatments of viral hemorrhagic fevers: A systematic review
Source: PLoS Negl Trop Dis. 2022 Oct 31;16(10):e0010889. doi: 10.1371/journal.pntd.0010889 (PMC9648854; doi:10.1371/journal.pntd.0010889)
Supplement: S5 Table — (DOCX) [file pntd.0010889.s006.docx]

S5 Table. Quality Assessment of studies reporting number of cases and/or case fatality rates

| **Study** | **VHF** | **Selection** | | | | **Comparability** | **Outcome** | | | | **Quality rating** |
| --- | --- | --- | --- | --- | --- | --- | --- | --- | --- | --- | --- |
|  |  | **Representativeness of the exposed cohort** | **Selection of the non-exposed cohort** | **Ascertainment of exposure** | **Demonstration that outcome of interest was not present at start of study** | **Comparability of cohorts on the basis of the design or analysis controlled for confounders** | **Assessment of outcome** | **Was follow-up long enough for outcomes to occur** | **Median duration of follow-up** | **Adequacy of follow-up of cohorts** |  |
| Adedire 2015 [1] | EVD | B | C | A | B | B | B | A | 3 months | B | Poor quality |
| Aguilar 2009 [2] | BHF | D | C | D | B | C | B | B | 1 year | B | Poor quality |
| Al-Abri 2019 [3] | CCHF | B | A | A | B | A | B | B | 22 years | B | Fair quality |
| Alonso 2019 [4] | HPS | B | C | E | B | A | B | B | 8 years | B | Fair quality |
| Archer 2013 [5] | RVF | B | A | A | B | C | B | B | 4 years | B | Fair quality |
| Argentinian website 2019 [6] | ArHF | B | C | E | B | B | B | A | 28 years | B | Poor quality |
| Aylward 2014 [7] | EVD | D | C | D | B | C | B | B | 6 months | B | Poor quality |
| Baba 2014 [8] | RVF | B | C | D | B | B | B | A | 3 years | B | Poor quality |
| Baba 2015 [9] | RVF | B | C | D | B | B | B | A | 3 years | B | Poor quality |
| Bangura 2009 [10] | LF | B | C | E | B | B | B | A | 5 years | B | Poor quality |
| Bausch 2001 [11] | LF | B | C | A | B | B | B | A | 4 years | B | Fair quality |
| Bayard 2004 [12] | HPS | B | C | A | B | B | B | A | 2 months | B | Poor quality |
| Bosch 2004 [13] | EVD | C | A | A | B | A | B | B | 26 years | B | Poor quality |
| Buba 2018 [14] | LF | B | C | D | B | B | B | A | 5 months | B | Poor quality |
| Butler 2014 [15] | EVD | B | A | A | B | B | B | B | 38 years | B | Poor quality |
| CDC 2014 [16] | EVD | B | A | A | B | C | B | A | 6 months | B | Poor quality |
| CDC 2014 [17] | EVD | B | A | A | B | B | B | B | 2 months | B | Poor quality |
| CDC 2014 [18] | EVD | B | A | A | A | B | B | A | 1 month | A | Poor quality |
| CDC 2014 [19] | EVD | B | A | A | A | B | B | B | 4 months | B | Poor quality |
| Chen 1993 [20] | HFRS | B | C | E | B | B | B | A | 59 years | B | Fair quality |
| Chérif 2017 [21] | EVD | B | C | D | B | B | B | A | 1 year | B | Poor quality |
| Chinikar 2010 [22] | CCHF | B | C | A | B | B | B | A | 9 years | B | Fair quality |
| Chinikar 2012 [23] | CCHF | B | C | E | B | B | B | A | 11 years | B | Fair quality |
| da Rosa Elkhoury 2012 [24] | HPS | B | C | A | B | B | B | A | 13 years | B | Poor quality |
| Dan-Nwafor 2019 [25] | LF | B | C | A | B | B | B | A | 4 months | B | Fair quality |
| Deng 1978 [26] | EVD | C | C | A | B | B | B | A | 4 months | B | Poor quality |
| Dixon 2014 [27] | EVD | C | C | A | A | B | B | A | 14 years | B | Poor quality |
| Duran 2013 [28] | CCHF | B | A | A | B | B | B | B | 6 months | B | Fair quality |
| Ellwanger 2017 [29] | SHF | B | C | E | B | B | B | A | 27 years | B | Poor quality |
| Eurosurveillance 2006 [30] | CCHF | B | B | A | B | B | B | B | 6 months | B | Fair quality |
| Gledovic 2008 [31] | HFRS | B | C | E | B | B | B | A | 10 years | B | Poor quality |
| Grobbelaar 2014 [32] | CCHF | B | A | D | B | B | B | B | 32 years | B | Fair quality |
| Hassan 2011 [33] | RVF | B | C | A | B | B | B | A | 1 year | B | Fair quality |
| Heyman 2007 [34] | HFRS | B | C | A | B | B | B | A | 1 year | B | Fair quality |
| Kateh 2015 [35] | EVD | B | C | A | B | B | B | A | 3 months | B | Poor quality |
| Khan 1996 [36] | HPS | B | C | A | B | B | B | A | 1 year | B | Fair quality |
| Khan 1999 [37] | EVD | B | C | E | B | B | B | A | 5 years | B | Poor quality |
| Klein 2011 [38] | HFRS | B | C | E | B | B | B | A | 5 years | B | Poor quality |
| Knust 2011 [39] | CCHF | B | A | A | B | B | B | B | 1 year | B | Poor quality |
| Kucharski 2014 [40] | EVD | B | C | D | B | B | B | A | 1 year | B | Poor quality |
| Lagare 2019 [41] | RVF | B | C | A | B | C | B | B | 5 months | B | Poor quality |
| Lee 2013 [42] | HFRS | B | C | A | B | B | B | A | 9 years | B | Poor quality |
| MacNeil 2010 [43] | EVD | B | C | A | B | B | B | A | 1 year | B | Poor quality |
| MacNeil 2011 [44] | HPS | B | C | A | B | B | B | A | 18 years | B | Poor quality |
| Madani 2003 [45] | RVF | B | C | A | B | B | B | A | 2 years | B | Fair quality |
| Madani 2011 [46] | AHF | B | C | D | B | B | B | A | 7 years | B | Fair quality |
| Majeed 2012 [47] | CCHF | B | C | D | B | B | B | A | 20 years | B | Fair quality |
| Makary 2010 [48] | HFRS | B | C | D | B | B | B | A | 13 years | B | Poor quality |
| Martinez 2010 [49] | HPS | B | C | E | B | B | B | A | 13 years | B | Fair quality |
| Melnik 2016 [50] | CCHF | B | C | E | B | B | B | A | 10 years | B | Poor quality |
| Memish 2014 [51] | AHF | B | C | D | B | B | B | A | 3 years | B | Fair quality |
| Mofleh 2012 [52] | CCHF | B | C | D | B | B | B | A | 4 months | B | Poor quality |
| Mohamed 2010 [53] | RVF | B | C | E | B | B | B | A | 6 months | B | Fair quality |
| Mulic 2002 [54] | HFRS | B | C | D | B | B | B | A | 14 years | B | Poor quality |
| Nabeth 2004 [55] | CCHF | B | C | D | B | B | B | A | 7 months | B | Poor quality |
| Nanclares 2016 [56] | EVD | B | A | E | B | C | B | B | 38 years | B | Poor quality |
| Nguku 2010 [57] | RVF | B | C | A | B | B | B | A | 1 year | B | Poor quality |
| Nkoghe 2005 [58] | EVD | B | C | D | B | B | B | A | 1 year | B | Poor quality |
| Nurmakhanov 2015 [59] | CCHF | B | C | D | B | B | B | A | 65 years | B | Fair quality |
| Nyenswah 2014 [60] | EVD | B | C | A | B | B | B | A | 6 months | B | Fair quality |
| Okware 2002 [61] | EVD | B | C | A | B | B | B | A | 1 year | B | Poor quality |
| PHLS 1996 [62] | EVD | B | A | A | B | B | B | B | 4 months | B | Poor quality |
| Pinto 2014 [63] | HPS | B | C | E | B | B | B | A | 10 years | B | Poor quality |
| Ratkovic 2016 [64] | HFRS | B | C | A | B | B | B | A | 10 years | B | Fair quality |
| Riquelme 2015 [65] | HPS | B | C | D | B | B | B | A | 17 years | B | Poor quality |
| Rosello 2015 [66] | EVD | B | A | A | B | B | B | B | 38 years | B | Fair quality |
| Růžek 2010 [67] | OHF | B | C | E | B | B | B | A | 52 years | B | Poor quality |
| Sahak 2019 [68] | CCHF | B | C | D | B | B | B | A | 3 years | B | Poor quality |
| Shiyou 2019 [69] | HFRS | B | C | D | B | B | B | A | 35 years | B | Poor quality |
| Tabatabaei 2014 [70] | CCHF | B | C | A | B | B | B | A | 13 years | B | Fair quality |
| Team WHO 2016 [71] | EVD | B | C | A | B | B | B | A | 2 years | B | Poor quality |
| Tkachenko 2019 [72] | HFRS | C | C | A | B | B | B | A | 17 years | B | Fair quality |
| Tumturk 2019 [73] | CCHF | B | B | A | B | B | B | A | 5 years | B | Poor quality |
| Venezuelan website 2012 [74] | VeHF | B | C | E | B | B | B | A | 21 years | B | Poor quality |
| Vescio 2012 [75] | CCHF | B | C | A | B | B | B | A | 12 years | B | Poor quality |
| WHO 1997 [76] | EVD | B | A | E | B | C | B | A | 6 months | B | Fair quality |
| WHO 1998 [77] | RVF | B | A | A | B | B | B | B | 1 year | B | Fair quality |
| WHO 2015 [78] | EVD | B | B | A | B | C | B | B | 1 year | B | Poor quality |
| Wilson 2014 [79] | ArHF, LF, LHF, VeHF | B | C | D | B | B | B | A | Multiple | B | Poor quality |
| Wong 2016 [80] | EVD | B | C | E | B | B | B | A | 9 months | B | Poor quality |
| World Health Organization 2005 [81] | MVD | B | C | D | B | B | B | A | 1 year | B | Poor quality |
| Ye 2021 [82] | HFRS | B | C | A | B | B | B | A | 4 years | B | Fair quality |
| Yesilyurt 2011 [83] | CCHF | C | C | A | B | B | B | A | 2 years | B | Poor quality |
| Yilmaz 2009 [84] | CCHF | B | C | E | B | B | B | A | 6 years | B | Poor quality |
| Zhang 2010 [85] | HFRS | C | C | A | B | B | B | A | 27 years | B | Poor quality |
| Zhang 2010 [86] | HFRS | B | C | E | B | B | B | A | 57 years | B | Poor quality |
| Zhang 2014 [87] | HFRS | B | C | D | B | B | B | A | 6 years | B | Poor quality |
| Zheng 2019 [88] | HFRS | C | C | E | B | B | B | A | 4 years | B | Poor quality |

*Note: AHF, Alkhurma hemorrhagic fever; ArHF, Argentine hemorrhagic fever; BHF, Bolivian hemorrhagic fever; CHF, Chapare hemorrhagic fever; CCHF, Crimean-Congo hemorrhagic fever; EVD, Ebola Virus Disease; HPS, Hantavirus Pulmonary Syndrome; HFRS, Hemorrhagic fever with renal syndrome; LF, Lassa fever; LHF, Lujo hemorrhagic fever; MVD, Marburg virus disease; OHF, Omsk hemorrhagic fever; RVF, Rift Valley fever; SHF, Sabia hemorrhagic fever; VeHF, Venezuelan hemorrhagic fever.*

References

1. Adedire E.B., Fatiregun A., Olayinka A., Sabitu K., Nguku P. Descriptive epidemiology of the EBOLA virus disease outbreak in Nigeria, July to September 2014. Am J Trop Med Hyg. 2015;93(4):55–6.

2. Aguilar PV, Camargo W, Vargas J, Guevara C, Roca Y, Felices V, et al. Reemergence of Bolivian hemorrhagic fever, 2007-2008. Emerg Infect Dis. 2009 Sep;15(9):1526–8.

3. Al-Abri SS, Hewson R, Al-Kindi H, Al-Abaidani I, Al-Jardani A, Al-Maani A, et al. Clinical and molecular epidemiology of Crimean-Congo hemorrhagic fever in Oman. PLoS Negl Trop Dis. 2019 Apr 25;13(4):e0007100–e0007100.

4. Alonso D., Iglesias A., Coelho R., Periolo N., Bruno A., Córdoba M., et al. Epidemiological description, case-fatality rate, and trends of Hantavirus Pulmonary Syndrome: 9 years of surveillance in Argentina. J Med Virol. 2019;91(7):1173–81.

5. Archer BN, Thomas J, Weyer J, Cengimbo A, Landoh DE, Jacobs C, et al. Epidemiologic Investigations into Outbreaks of Rift Valley Fever in Humans, South Africa, 2008-2011. Emerg Infect Dis. 2013 Dec;19(12):1918–25.

6. Sociedad Argentina de Vacunología y Epidemiologia, Sociedad Argentina de Virología, Subcomisión Vacunología de la Asociación Argentina de Microbiología. FIEBRE HEMORRÁGICA ARGENTINA [Internet]. 2019. Available from: https://save.org.ar/wp-content/uploads/2019/07/Documento-Posicion-Fiebre-Hemorragica-Argentina-.pdf

7. Aylward B., Barboza P., Bawo L., Bertherat E., Bilivogui P., Blake I., et al. Ebola virus disease in West Africa - The first 9 months of the epidemic and forward projections. N Engl J Med. 2014;371(16):1481–95.

8. Baba M.M., Masiga D., Villinger J. Cyclical outbreaks of rift valley fever in East Africa: Why they persist and possible solutions to prevent or contain its spread. Am J Trop Med Hyg. 2014;91(5):260.

9. Baba M.M., Villinger J., Sang R., Masiga D. Has rift valley fever virus evolved to increased virulence in human population after existence for a century in east Africa? Am J Trop Med Hyg. 2015;93(4):201.

10. Bangura J.J., Fair J., Goba A., Khan S.H., Fonnie R., Garry R.F., et al. Epidemiology of lassa fever in the mano river union countries of West Africa, 2004-2008. Am J Trop Med Hyg. 2009;81(5):209–10.

11. Bausch D.G., Demby A.H., Coulibaly M., Kanu J., Goba A., Bah A., et al. Lassa fever in Guinea: I. Epidemiology of human disease and clinical observations. Vector Borne Zoonotic Dis Larchmt N. 2001;1(4):269–81.

12. Bayard V, Kitsutani PT, Barria EO, Ruedas LA, Tinnin DS, Muñoz C, et al. Outbreak of hantavirus pulmonary syndrome, Los Santos, Panama, 1999-2000. Emerg Infect Dis. 2004 Sep;10(9):1635–42.

13. Bosch X. Sudan Ebola outbreak of known strain. Lancet Infect Dis. 2004 Jul;4(7):388–388.

14. Buba MI, Dalhat MM, Nguku PM, Waziri N, Mohammad JO, Bomoi IM, et al. Mortality Among Confirmed Lassa Fever Cases During the 2015-2016 Outbreak in Nigeria. Am J Public Health. 2018 Feb;108(2):262–4.

15. Butler D, Morello L. Ebola by the numbers: The size, spread and cost of an outbreak. Nature. 2014 Oct 16;514(7522):284–5.

16. Incident Management System Ebola Epidemiology Team CM of H of G Sierra Leone, Liberia, Nigeria, and Senegal; Viral Special Pathogens Branch, National Center for Emerging and Zoonotic Infectious Diseases, CDC. Ebola virus disease outbreak - West Africa, September 2014. MMWR Morb Mortal Wkly Rep. 2014 Oct 3;63(39):865–6.

17. Incident Management System Ebola Epidemiology Team C, Guinea Interministerial Committee for Response Against the Ebola Virus; CDC Guinea Response Team, Liberia Ministry of Health and Social Welfare, CDC Liberia Response Team, Sierra Leone Ministry of Health and Sanitation, CDC Sierra Leone Response Team, et al. Update: Ebola virus disease outbreak--West Africa, October 2014. MMWR Morb Mortal Wkly Rep. 2014 Oct 31;63(43):978–81.

18. Incident Management System Ebola Epidemiology Team C, Guinea Interministerial Committee for Response Against the Ebola Virus, World Health Organization, CDC Guinea Response Team, Liberia Ministry of Health and Social Welfare, CDC Liberia Response Team, et al. Update: Ebola virus disease epidemic--West Africa, November 2014. MMWR Morb Mortal Wkly Rep. 2014 Nov 21;63(46):1064–6.

19. Incident Management System Ebola Epidemiology Team, Guinea Interministerial Committee for Response Against the Ebola Virus, World Health Organization, CDC Guinea Response Team; Liberia Ministry of Health and Social Welfare, CDC Liberia Response Team; Sierra Leone Ministry of Health and Sanitation, CDC Sierra Leone Response Team, et al. Update: ebola virus disease epidemic - West Africa, December 2014. MMWR Morb Mortal Wkly Rep. 2014 Dec 19;63(50):1199–201.

20. Chen HX, Qiu FX. Epidemiologic surveillance on the hemorrhagic fever with renal syndrome in China. Chin Med J (Engl). 1993 Nov;106(11):857–63.

21. Chérif MS, Koonrungsesomboon N, Kassé D, Cissé SD, Diallo SB, Chérif F, et al. Ebola virus disease in children during the 2014-2015 epidemic in Guinea: a nationwide cohort study. Eur J Pediatr. 2017 Jun;176(6):791–6.

22. Chinikar S, Ghiasi SM, Moradi M, Goya MM, Shirzadi MR, Zeinali M, et al. Geographical distribution and surveillance of Crimean-Congo hemorrhagic fever in Iran. Vector Borne Zoonotic Dis Larchmt N. 2010 Oct;10(7):705–8.

23. Chinikar S., Moradi M., Khakifirouz S., Rasi Varaie F.S., Rafigh M., Hassanzehi A. Last situation of Crimean-Congo haemorrhagic fever in Iran and its public health importance. Clin Microbiol Infect. 2012;18:716.

24. da Rosa Elkhoury M, da Silva Mendes W, Waldman EA, Dias JP, Carmo EH, Fernando da Costa Vasconcelos P. Hantavirus pulmonary syndrome: prognostic factors for death in reported cases in Brazil. Trans R Soc Trop Med Hyg. 2012 May;106(5):298–302.

25. Dan-Nwafor C.C., Furuse Y., Ilori E.A., Ipadeola O., Akabike K.O., Ahumibe A., et al. Measures to control protracted large lassa fever outbreak in Nigeria, 1 January to 28 April 2019. Eurosurveillance. 2019;24(20).

26. Deng I.M., Duku O., Gillo A.L. Ebola haemorrhagic fever in Sudan, 1976. Report of a WHO/International Study Team. Bull World Health Organ. 1978;56(2):247–70.

27. Dixon MG, Schafer IJ, Centers for Disease Control and Prevention (CDC). Ebola viral disease outbreak--West Africa, 2014. MMWR Morb Mortal Wkly Rep. 2014 Jun 27;63(25):548–51.

28. Duran A, Küçükbayrak A, Ocak T, Hakyemez NI, Taþ T, Karadađ M, et al. Evaluation of patients with Crimean-Congo hemorrhagic fever in Bolu, Turkey. Afr Health Sci. 2013 Jun;13(2):233–42.

29. Ellwanger JH, Chies JAB. Keeping track of hidden dangers - The short history of the Sabiá virus. Rev Soc Bras Med Trop. 2017 Feb;50(1):3–8.

30. Increase in cases of Crimean-Congo haemorrhagic fever, Turkey, 2006. Euro Surveill Bull Eur Sur Mal Transm Eur Commun Dis Bull. 2006 Jul 20;11(7):E060720.2-E060720.2.

31. Gledovic ZB, Jeknic AS, Grgurevic AD, Rakocevic BB, Bozovic BR, Mugosa BV. Hemorrhagic fever with renal syndrome in Montenegro. Jpn J Infect Dis. 2008 Sep;61(5):386–7.

32. Grobbelaar A.A., Weyer J., Msimang V., Kemp A., Paweska J.T. Epidemiologic and phylogenetic characteristics of Crimean-Congo haemorrhagic fever in South Africa, 1981-2013. Int J Infect Dis. 2014;21:191.

33. Hassan OA, Ahlm C, Sang R, Evander M. The 2007 Rift Valley fever outbreak in Sudan. PLoS Negl Trop Dis. 2011 Sep;5(9):e1229.

34. Heyman P, Cochez C, Ducoffre G, Mailles A, Zeller H, Abu Sin M, et al. Haemorrhagic Fever with Renal Syndrome: an analysis of the outbreaks in Belgium, France, Germany, the Netherlands and Luxembourg in 2005. Euro Surveill Bull Eur Sur Mal Transm Eur Commun Dis Bull. 2007 May 1;12(5):E15-16.

35. Kateh F, Nagbe T, Kieta A, Barskey A, Gasasira AN, Driscoll A, et al. Rapid response to Ebola outbreaks in remote areas - Liberia, July-November 2014. MMWR Morb Mortal Wkly Rep. 2015 Feb 27;64(7):188–92.

36. Khan A.S., Khabbaz R.F., Armstrong L.R., Holman R.C., Bauer S.P., Graber J., et al. Hantavirus pulmonary syndrome: The first 100 US cases. J Infect Dis. 1996;173(6):1297–303.

37. Khan A.S., Tshioko F.K., Heymann D.L., Le Guenno B., Nabeth P., Kerstiëns B., et al. The reemergence of Ebola hemorrhagic fever, Democratic Republic of the Congo, 1995. J Infect Dis. 1999;179(SUPPL. 1):S76–86.

38. Klein SL, Marks MA, Li W, Glass GE, Fang LQ, Ma JQ, et al. Sex differences in the incidence and case fatality rates from hemorrhagic fever with renal syndrome in China, 2004-2008. Clin Infect Dis Off Publ Infect Dis Soc Am. 2011 Jun 15;52(12):1414–21.

39. Knust B., MacNeil A., Erickson B.R., Medetov Z.B., Ospanov K.S. Crimean congo hemorrhagic fever surveillance in Kazakhstan, 2009-2010. Am J Trop Med Hyg. 2011;85(6):282.

40. Kucharski A.J., Edmunds W.J. Case fatality rate for Ebola virus disease in West Africa. The Lancet. 2014;384(9950):1260.

41. Lagare A., Fall G., Ibrahim A., Ousmane S., Sadio B., Abdoulaye M., et al. First occurrence of Rift Valley fever outbreak in Niger, 2016. Vet Med Sci. 2019;5(1):70–8.

42. Lee SH, Chung BH, Lee WC, Choi IS. Epidemiology of hemorrhagic fever with renal syndrome in Korea, 2001-2010. J Korean Med Sci. 2013 Oct;28(10):1552–4.

43. MacNeil A, Farnon EC, Wamala J, Okware S, Cannon DL, Reed Z, et al. Proportion of deaths and clinical features in Bundibugyo Ebola virus infection, Uganda. Emerg Infect Dis. 2010 Dec;16(12):1969–72.

44. MacNeil A, Ksiazek TG, Rollin PE. Hantavirus pulmonary syndrome, United States, 1993-2009. Emerg Infect Dis. 2011 Jul;17(7):1195–201.

45. Madani TA, Al-Mazrou YY, Al-Jeffri MH, Mishkhas AA, Al-Rabeah AM, Turkistani AM, et al. Rift Valley fever epidemic in Saudi Arabia: epidemiological, clinical, and laboratory characteristics. Clin Infect Dis Off Publ Infect Dis Soc Am. 2003 Oct 15;37(8):1084–92.

46. Madani TA, Azhar EI, Abuelzein ETME, Kao M, Al-Bar HMS, Abu-Araki H, et al. Alkhumra (Alkhurma) virus outbreak in Najran, Saudi Arabia: epidemiological, clinical, and laboratory characteristics. J Infect. 2011 Jan;62(1):67–76.

47. Majeed B., Dicker R., Nawar A., Badri S., Noah A., Muslem H. Morbidity and mortality of Crimean-Congo hemorrhagic fever in Iraq: Cases reported to the National Surveillance System, 1990-2010. Trans R Soc Trop Med Hyg. 2012;106(8):480–3.

48. Makary P, Kanerva M, Ollgren J, Virtanen MJ, Vapalahti O, Lyytikäinen O. Disease burden of Puumala virus infections, 1995-2008. Epidemiol Infect. 2010 Oct;138(10):1484–92.

49. Martinez VP, Bellomo CM, Cacace ML, Suarez P, Bogni L, Padula PJ. Hantavirus pulmonary syndrome in Argentina, 1995-2008. Emerg Infect Dis. 2010 Dec;16(12):1853–60.

50. Melnik V., Rakhmanova N.A., Pshenichnaya N., Aslanov B. Crimean-Congo Hemorrhagic fever in former Soviet Union countries based on ProMED-RUS reports (2005-2015 years). Int J Infect Dis. 2016;45:183.

51. Memish ZA, Fagbo SF, Osman Ali A, AlHakeem R, Elnagi FM, Bamgboye EA. Is the epidemiology of alkhurma hemorrhagic fever changing?: A three-year overview in Saudi Arabia. PloS One. 2014 Feb 6;9(2):e85564–e85564.

52. Mofleh J, Ahmad Z. Crimean-Congo haemorrhagic fever outbreak investigation in the Western Region of Afghanistan in 2008. East Mediterr Health J Rev Sante Mediterr Orient Al-Majallah Al-Sihhiyah Li-Sharq Al-Mutawassit. 2012 May;18(5):522–6.

53. Mohamed M, Mosha F, Mghamba J, Zaki SR, Shieh WJ, Paweska J, et al. Epidemiologic and clinical aspects of a Rift Valley fever outbreak in humans in Tanzania, 2007. Am J Trop Med Hyg. 2010 Aug;83(2 Suppl):22–7.

54. Mulić R, Ropac D, R. M, D. R. Epidemiologic characteristics and military implications of hemorrhagic fever with renal syndrome in Croatia. Croat Med J. 2002 Oct;43(5):581–6.

55. Nabeth P, Cheikh DO, Lo B, Faye O, Vall IOM, Niang M, et al. Crimean-Congo hemorrhagic fever, Mauritania. Emerg Infect Dis. 2004 Dec;10(12):2143–9.

56. Nanclares C., Kapetshi J., Lionetto F., De La Rosa O., Muyembe Tamfun J.-J., Alia M., et al. Ebola virus disease, democratic republic of the Congo, 2014. Emerg Infect Dis. 2016;22(9):1579–86.

57. Nguku PM, Sharif SK, Mutonga D, Amwayi S, Omolo J, Mohammed O, et al. An investigation of a major outbreak of Rift Valley fever in Kenya: 2006-2007. Am J Trop Med Hyg. 2010 Aug;83(2 Suppl):5–13.

58. Nkoghe D., Formenty P., Leroy E.M., Nnegue S., Edou S.Y., Ba J.I., et al. Multiple Ebola virus haemorrhagic fever outbreaks in Gabon, from October 2001 to April 2002. Bull Société Pathol Exot 1990. 2005;98(3):224–9.

59. Nurmakhanov T, Sansyzbaev Y, Atshabar B, Deryabin P, Kazakov S, Zholshorinov A, et al. Crimean-Congo haemorrhagic fever virus in Kazakhstan (1948-2013). Int J Infect Dis IJID Off Publ Int Soc Infect Dis. 2015 Sep;38:19–23.

60. Nyenswah T, Fahnbulleh M, Massaquoi M, Nagbe T, Bawo L, Falla JD, et al. Ebola epidemic--Liberia, March-October 2014. MMWR Morb Mortal Wkly Rep. 2014 Nov 21;63(46):1082–6.

61. Okware SI, Omaswa FG, Zaramba S, Opio A, Lutwama JJ, Kamugisha J, et al. An outbreak of Ebola in Uganda. Trop Med Int Health TM IH. 2002 Dec;7(12):1068–75.

62. Outbreak of Ebola haemorrhagic fever in Gabon. Commun Dis Rep CDR Wkly. 1996 Mar 1;6(9):75–8.

63. Pinto VL, DE Sousa AI, DE Lemos ERS. Regional variations and time trends of hantavirus pulmonary syndrome in Brazil. Epidemiol Infect. 2014 Oct;142(10):2166–71.

64. Ratkovic M., Basic-Jukic N., Gledovic B., Radunovic D., Prelevic V. Winter time hemorrhagic fever with renal syndrome in Montenegro-new challenge. Nephrol Dial Transplant. 2016;31:i153–4.

65. Riquelme R, Rioseco ML, Bastidas L, Trincado D, Riquelme M, Loyola H, et al. Hantavirus pulmonary syndrome, Southern Chile, 1995-2012. Emerg Infect Dis. 2015 Apr;21(4):562–8.

66. Rosello A, Mossoko M, Flasche S, Van Hoek AJ, Mbala P, Camacho A, et al. Ebola virus disease in the Democratic Republic of the Congo, 1976-2014. eLife. 2015 Nov 3;4:e09015.

67. Růžek D, Yakimenko VV, Karan LS, Tkachev SE. Omsk haemorrhagic fever. Lancet Lond Engl. 2010 Dec 18;376(9758):2104–13.

68. Sahak MN, Arifi F, Saeedzai SA. Descriptive epidemiology of Crimean-Congo Hemorrhagic Fever (CCHF) in Afghanistan: Reported cases to National Surveillance System, 2016-2018. Int J Infect Dis IJID Off Publ Int Soc Infect Dis. 2019 Nov;88:135–40.

69. Liu S, Wei Y, Han X, Cai Y, Han Z, Zhang Y, et al. Long-term retrospective observation reveals stabilities and variations of hantavirus infection in Hebei, China. BMC Infect Dis. 2019 Sep 2;19(1):765–765.

70. Tabatabaei S.M., Hassanzehi A. Crimean Congo hemorrhagic fever case fatality and associated factors in southeast of Iran, 1999-2012. Int J Infect Dis. 2014;21:240.

71. WHO Ebola Response Team, Agua-Agum J, Ariyarajah A, Blake IM, Cori A, Donnelly CA, et al. Ebola Virus Disease among Male and Female Persons in West Africa. N Engl J Med. 2016 Jan 7;374(1):96–8.

72. Tkachenko E.A., Ishmukhametov A.A., Dzagurova T.K., Bernshtein A.D., Morozov V.G., Siniugina A.A., et al. Hemorrhagic fever with renal syndrome, Russia. Emerg Infect Dis. 2019;25(12):2325–8.

73. Tumturk A. Crimean-Congo haemorrhagic fever in a middle Anatolian city: five years of experience. Trop Doct. 2019;

74. Paredes Varga H. Fiebre hemorrágica venezolana [Internet]. 2012. Available from: https://botica.xyz/24/

75. Vescio FM, Busani L, Mughini-Gras L, Khoury C, Avellis L, Taseva E, et al. Environmental correlates of Crimean-Congo haemorrhagic fever incidence in Bulgaria. BMC Public Health. 2012 Dec 27;12:1116–1116.

76. Ebola haemorrhagic fever. A summary of the outbreak in Gabon. Releve Epidemiol Hebd. 1997 Jan 3;72(1–2):7–8.

77. An outbreak of Rift Valley Fever, eastern Africa, 1997-1998. Releve Epidemiol Hebd. 1998 Apr 10;73(15):105–9.

78. Ebola virus disease (EVD) in West Africa: an extraordinary epidemic. Releve Epidemiol Hebd. 2015 Mar 6;90(10):89–96.

79. Wilson M.R., Peters C.J. Diseases of the central nervous system caused by lymphocytic choriomeningitis virus and other arenaviruses. Handb Clin Neurol. 2014;123((Wilson M.R., michaelneuro@gmail.com) Multiple Sclerosis Center, Department of Neurology, School of Medicine, University of California San Francisco, San Francisco, CA, United States):671–81.

80. Wong JY, Zhang W, Kargbo D, Haque U, Hu W, Wu P, et al. Assessment of the severity of Ebola virus disease in Sierra Leone in 2014-2015. Epidemiol Infect. 2016 May;144(7):1473–81.

81. World Health Organization. Marburg haemorrhagic fever, Angola--update. Releve Epidemiol Hebd. 2005 Apr 22;80(16):141–2.

82. Ye GH, Alim M, Guan P, Huang DS, Zhou BS, Wu W. Improving the precision of modeling the incidence of hemorrhagic fever with renal syndrome in mainland China with an ensemble machine learning approach. PloS One. 2021;16(3):e0248597.

83. Yesilyurt M, Gul S, Ozturk B, Kayhan BC, Celik M, Uyar C, et al. The early prediction of fatality in Crimean Congo hemorrhagic fever patients. Saudi Med J. 2011 Jul;32(7):742–3.

84. Yilmaz R, Kundak AA, Ozer S, Esmeray H. Successful treatment of severe Crimean-Congo hemorrhagic fever with supportive measures without ribavirin and hypothermia. J Clin Virol Off Publ Pan Am Soc Clin Virol. 2009 Feb;44(2):181–2.

85. Zhang YZ, Lin XD, Shi NF, Wang W, Liao XW, Guo WP, et al. Hantaviruses in small mammals and humans in the coastal region of Zhejiang Province, China. J Med Virol. 2010 May;82(6):987–95.

86. Zhang YZ, Zou Y, Fu ZF, Plyusnin A. Hantavirus infections in humans and animals, China. Emerg Infect Dis. 2010 Aug;16(8):1195–203.

87. Zhang S, Wang S, Yin W, Liang M, Li J, Zhang Q, et al. Epidemic characteristics of hemorrhagic fever with renal syndrome in China, 2006-2012. BMC Infect Dis. 2014 Jul 11;14:384–384.

88. Zheng Z, Wang P, Wang Z, Zhang D, Wang X, Zuo S, et al. The characteristics of current natural foci of hemorrhagic fever with renal syndrome in Shandong Province, China, 2012-2015. PLoS Negl Trop Dis. 2019 May 20;13(5):e0007148–e0007148.
