# Supplementary material for: The number of cases, mortality and treatments of viral hemorrhagic fevers: A systematic review
Source: PLoS Negl Trop Dis. 2022 Oct 31;16(10):e0010889. doi: 10.1371/journal.pntd.0010889 (PMC9648854; doi:10.1371/journal.pntd.0010889)
Supplement: S6 Table — (DOCX) [file pntd.0010889.s007.docx]

S6 Table. Number of cases and CFRs of Crimean-Congo hemorrhagic fever by country (alphabetical order) and period

| **Country** | **Period** | **Number of cases** | **Case fatality rate** | **Case definition** |
| --- | --- | --- | --- | --- |
| Afghanistan |  |  |  |  |
|  | 2008 | 30 | 33% | Confirmed cases |
|  | 2016 -2018 | 293 | 43% | Confirmed cases |
| Bulgaria |  |  |  |  |
|  | 1997 -2009 | 159 | 26% | Confirmed cases |
| Georgia |  |  |  |  |
|  | 2012 -2015 | 36 | 20% | Not specified |
| Iran |  |  |  |  |
|  | 2000 – 2009 | 635 | 14% | Confirmed cases |
|  | 2000 – 2011 | 853 | 14% | Confirmed cases |
| Iraq |  |  |  |  |
|  | 1990 - 1997 | 271 | NR | Confirmed cases |
|  | 1997 -2009 | 32 | NR | Confirmed cases |
|  | 2010 | 11 | 36% | Confirmed cases |
| Kazakhstan |  |  |  |  |
|  | 1948 – 1969 | 89 | 25% | Confirmed cases |
|  | 1970 – 1979 | 33 | 31% | Confirmed cases |
|  | 1980 – 1989 | 148 | 21% | Confirmed cases |
|  | 1990 – 1999 | 222 | 11% | Confirmed cases |
|  | 2000 – 2009 | 162 | 14% | Confirmed cases |
|  | 2010 – 2013 | 50 | 14% | Confirmed cases |
| Kosovo |  |  |  |  |
|  | 2001 | 18 | 33% | Confirmed cases |
| Mauritania |  |  |  |  |
|  | 2003 | 34 | NR | Confirmed cases |
| Oman |  |  |  |  |
|  | 1995 – 2017 | 88 | 36% | Confirmed cases |
| Pakistan |  |  |  |  |
|  | 2010 | 26 | 12% | Not specified |
| South Africa |  |  |  |  |
|  | 1981 – 2013 | 193 | 24% | Confirmed cases |
| Tajikistan |  |  |  |  |
|  | 2009 | 5 | 60% | Not specified |
| Turkey |  |  |  |  |
|  | 2003 | 150 | 4% | Confirmed cases |
|  | 2004 | 249 | 5% | Confirmed cases |
|  | 2005 | 166 | 5% | Confirmed cases |
|  | 2006 | 438 | 4% | Confirmed cases |
|  | 2007 | 717 | 5% | Confirmed cases |
|  | 2008 - 2010 | 352 | 5% | Not specified |
|  | 2011 - 2015 | 4466 | 5% | Not specified |
| Uzbekistan |  |  |  |  |
|  | 2013 - 2015 | 13 | 77% | Not specified |

*Note: NR, Not reported*
