# Supplementary material for: The number of cases, mortality and treatments of viral hemorrhagic fevers: A systematic review
Source: PLoS Negl Trop Dis. 2022 Oct 31;16(10):e0010889. doi: 10.1371/journal.pntd.0010889 (PMC9648854; doi:10.1371/journal.pntd.0010889)
Supplement: S7 Table — (DOCX) [file pntd.0010889.s008.docx]

S7 Table. Number of cases and CFRs of Ebola Virus Disease by country and period

| **Country** | **Period** | **Number of cases** | **Case fatality rate** | **Case definition** |
| --- | --- | --- | --- | --- |
| Democratic Republic of the Congo |  |  |  |  |
|  | 1976 | 318 | 88% | Not specified |
|  | 1977 | 1 | 100% | Not specified |
|  | 1995 | 315 | 81% | Confirmed cases |
|  | 2001-2002 | 59 | 75% | Probable and confirmed cases |
|  | 2003 | 178 | 88% | Not specified |
|  | 2005 | 12 | 83% | Not specified |
|  | 2007 | 24 | 71% | Confirmed cases |
|  | 2008-2009 | 10 | 20% | Confirmed cases |
|  | 2012 | 38 | 34% | Confirmed cases |
|  | 2014 | 38 | 55% | Confirmed cases |
|  | 2017 | 8 | 50% | Suspected cases |
|  | 2018 | 3470 | 66% | Not specified |
| Gabon |  |  |  |  |
|  | 1994 | 52 | 60% | Not specified |
|  | 1996 | 91 | 73% | Not specified |
|  | 2001-2002 | 65 | 82% | Probable and confirmed cases |
| Guinea |  |  |  |  |
|  | 2014-2016 | 3811 | 67% | Suspected, probable and confirmed cases |
| Liberia |  |  |  |  |
|  | 2014-2016 | 10678 | 45% | Suspected, probable and confirmed cases |
| Mali |  |  |  |  |
|  | 2014 | 8 | 75% | Not specified |
| Nigeria |  |  |  |  |
|  | 2014 | 20 | 40% | Probable and confirmed cases |
| Sierra Leone |  |  |  |  |
|  | 2014-2016 | 14124 | 28% | Suspected, probable and confirmed cases |
| Sudan |  |  |  |  |
|  | 1976 | 284 | 53% | Not specified |
|  | 1979 | 34 | 65% | Not specified |
|  | 2004 | 17 | 41% | Not specified |
| Uganda |  |  |  |  |
|  | 2000 | 425 | 53% | Suspected, probable and confirmed cases |
|  | 2007 | 149 | 25% | Not specified |
|  | 2011 | 1 | 100% | Not specified |
|  | 2012 | 17 | 41% | Confirmed cases |
