# Supplementary material for: The number of cases, mortality and treatments of viral hemorrhagic fevers: A systematic review
Source: PLoS Negl Trop Dis. 2022 Oct 31;16(10):e0010889. doi: 10.1371/journal.pntd.0010889 (PMC9648854; doi:10.1371/journal.pntd.0010889)
Supplement: S8 Table — (DOCX) [file pntd.0010889.s009.docx]

S8 Table. Number of cases and CFRs of Hantavirus Pulmonary Syndrome by country and period

| **Country** | **Period** | **Number of cases** | **Case fatality rate** | **Case definition** |
| --- | --- | --- | --- | --- |
| Argentina |  |  |  |  |
|  | 1995-2008 | 710 | 26% | Confirmed cases |
|  | 2009-2017 | 533 | 21% | Confirmed cases |
| Brazil |  |  |  |  |
|  | 1993-2006 | 884 | 39% | Not specified |
|  | 2001-2011 | 1486 | NR | Confirmed cases |
| Chile |  |  |  |  |
|  | 1995-2012 | 103 | 32% | Confirmed cases |
| Panama |  |  |  |  |
|  | 1999-2000 | 12 | 25% | Suspected and confirmed cases |
| Paraguay |  |  |  |  |
|  | 1995-1996 | 23 | 48% | Confirmed cases |
| USA |  |  |  |  |
|  | 1993 | 48 | 56% | Confirmed cases |
|  | 1994 | 32 | 38% | Confirmed cases |
|  | 1995 | 24 | 42% | Confirmed cases |
|  | 1996 | 21 | 29% | Confirmed cases |
|  | 1997 | 23 | 22% | Confirmed cases |
|  | 1998 | 33 | 27% | Confirmed cases |
|  | 1999 | 43 | 33% | Confirmed cases |
|  | 2000 | 46 | 24% | Confirmed cases |
|  | 2001 | 11 | 27% | Confirmed cases |
|  | 2002 | 23 | 43% | Confirmed cases |
|  | 2003 | 31 | 29% | Confirmed cases |
|  | 2004 | 27 | 19% | Confirmed cases |
|  | 2005 | 34 | 29% | Confirmed cases |
|  | 2006 | 41 | 41% | Confirmed cases |
|  | 2007 | 29 | 41% | Confirmed cases |
|  | 2008 | 24 | 50% | Confirmed cases |
|  | 2009 | 19 | 21% | Confirmed cases |
|  | 2010 | 20 | 25% | Confirmed cases |
|  | 2011 | 24 | 50% | Confirmed cases |
|  | 2012 | 30 | 40% | Confirmed cases |
|  | 2013 | 20 | 45% | Confirmed cases |
|  | 2014 | 34 | 38% | Confirmed cases |
|  | 2015 | 22 | 27% | Confirmed cases |
|  | 2016 | 37 | 35% | Confirmed cases |
|  | 2017 | 33 | 21% | Confirmed cases |
|  | 2018 | 3 | 67% | Confirmed cases |

*Note: NR, Not reported*
