# Supplementary material for: The number of cases, mortality and treatments of viral hemorrhagic fevers: A systematic review
Source: PLoS Negl Trop Dis. 2022 Oct 31;16(10):e0010889. doi: 10.1371/journal.pntd.0010889 (PMC9648854; doi:10.1371/journal.pntd.0010889)
Supplement: S9 Table — (DOCX) [file pntd.0010889.s010.docx]

S9 Table. Number of cases and CFRs of Hemorrhagic fever with renal syndrome by country and period

| **Country** | **Period** | **Number of cases** | **Case fatality rate** | **Case definition** |
| --- | --- | --- | --- | --- |
| China |  |  |  |  |
|  | 1931-1941 | 10000 | 30% | Confirmed cases |
|  | 1942 | 102 | 15% | Confirmed cases |
|  | 1950 | 18 | 0% | Confirmed cases |
|  | 1951 | 10 | 0% | Confirmed cases |
|  | 1952 | 12 | 8% | Confirmed cases |
|  | 1953 | 10 | 0% | Confirmed cases |
|  | 1954 | 16 | 6% | Confirmed cases |
|  | 1955 | 455 | 7% | Confirmed cases |
|  | 1956 | 122 | 14% | Confirmed cases |
|  | 1957 | 489 | 6% | Confirmed cases |
|  | 1958 | 866 | 10% | Confirmed cases |
|  | 1959 | 1570 | 8% | Confirmed cases |
|  | 1960 | 600 | 7% | Confirmed cases |
|  | 1961 | 2455 | 11% | Confirmed cases |
|  | 1962 | 3189 | 10% | Confirmed cases |
|  | 1963 | 2932 | 11% | Confirmed cases |
|  | 1964 | 3520 | 10% | Confirmed cases |
|  | 1965 | 3076 | 11% | Confirmed cases |
|  | 1966 | 3371 | 11% | Confirmed cases |
|  | 1967 | 1967 | 9% | Confirmed cases |
|  | 1968 | 1515 | 10% | Confirmed cases |
|  | 1969 | 1139 | 14% | Confirmed cases |
|  | 1970 | 3324 | 11% | Confirmed cases |
|  | 1971 | 7290 | 13% | Confirmed cases |
|  | 1972 | 12243 | 11% | Confirmed cases |
|  | 1973 | 18203 | 10% | Confirmed cases |
|  | 1974 | 15211 | 9% | Confirmed cases |
|  | 1975 | 18672 | 8% | Confirmed cases |
|  | 1976 | 15647 | 8% | Confirmed cases |
|  | 1977 | 17026 | 8% | Confirmed cases |
|  | 1978 | 15158 | 7% | Confirmed cases |
|  | 1979 | 21175 | 7% | Confirmed cases |
|  | 1980 | 30447 | 6% | Confirmed cases |
|  | 1981 | 51822 | 5% | Confirmed cases |
|  | 1982 | 61119 | 5% | Confirmed cases |
|  | 1983 | 85237 | 4% | Confirmed cases |
|  | 1984 | 90927 | 3% | Confirmed cases |
|  | 1985 | 103751 | 3% | Confirmed cases |
|  | 1986 | 115985 | 2% | Confirmed cases |
|  | 1987 | 65201 | 2% | Confirmed cases |
|  | 1988 | 51510 | 2% | Confirmed cases |
|  | 1989 | 40075 | 3% | Confirmed cases |
|  | 1990 | 37640 | 2% | Confirmed cases |
|  | 1997-2003 | 265692 | ~1% | Confirmed cases |
|  | 2004 | 26177 | ~1% | Confirmed cases |
|  | 2005 | 22288 | ~1% | Confirmed cases |
|  | 2006 | 15098 | 1% | Confirmed cases |
|  | 2007 | 11063 | 1% | Confirmed cases |
|  | 2008 | 9039 | 1% | Confirmed cases |
|  | 2009 | 8745 | 1% | Confirmed cases |
|  | 2010 | 9526 | 1% | Confirmed cases |
|  | 2011 | 10779 | 1% | Confirmed cases |
|  | 2012 | 13308 | 1% | Confirmed cases |
| Croatia |  |  |  |  |
|  | 1987 | 6 | 0% | Not specified |
|  | 1989 | 16 | 0% | Not specified |
|  | 1990 | 2 | 0% | Not specified |
|  | 1992 | 13 | 15% | Not specified |
|  | 1993 | 1 | 0% | Not specified |
|  | 1994 | 7 | 0% | Not specified |
|  | 1995 | 129 | 2% | Not specified |
|  | 1996 | 6 | 0% | Not specified |
|  | 1997 | 8 | 0% | Not specified |
|  | 1998 | 16 | 0% | Not specified |
|  | 1999 | 15 | 0% | Not specified |
|  | 2000 | 5 | 0% | Not specified |
|  | 2001 | 11 | 0% | Not specified |
| Finland |  |  |  |  |
|  | 1995-2008 | 22681 | ~0.1% | Confirmed cases |
| Belgium |  |  |  |  |
|  | 2005 | 372 | NR | Confirmed cases |
| France |  |  |  |  |
|  | 2005 | 253 | NR | Confirmed cases |
| Germany |  |  |  |  |
|  | 2005 | 448 | NR | Confirmed cases |
| Netherlands |  |  |  |  |
|  | 2005 | 27 | NR | Confirmed cases |
| Luxembourg |  |  |  |  |
|  | 2005 | 14 | NR | Confirmed cases |
| South Korea |  |  |  |  |
|  | 1951-1953 | 1411 | NR | Confirmed cases |
|  | 2001-2010 | 3953 | 1% | Confirmed cases |
| Montenegro |  |  |  |  |
|  | 1995-2005 | 169 | 5% | Confirmed cases |
| Russia |  |  |  |  |
|  | 2000-2017 | 131590 | 0.4% | Confirmed cases |

*Note: NR, Not reported*
