# Supplementary material for: The number of cases, mortality and treatments of viral hemorrhagic fevers: A systematic review
Source: PLoS Negl Trop Dis. 2022 Oct 31;16(10):e0010889. doi: 10.1371/journal.pntd.0010889 (PMC9648854; doi:10.1371/journal.pntd.0010889)
Supplement: S10 Table — (DOCX) [file pntd.0010889.s011.docx]

S10 Table. Number of cases and CFRs of Lassa fever by country and period

| **Country** | **Period** | **Number of cases** | **Case fatality rate** | **Case definition** |
| --- | --- | --- | --- | --- |
| Benin |  |  |  |  |
|  | 2016 | 54 | 52% | Suspected cases |
| Liberia |  |  |  |  |
|  | 2016-2018 | 33 | 45% | Confirmed cases |
| Nigeria |  |  |  |  |
|  | 2012 | 623 | 11% | Suspected cases |
|  | 2015-2016 | 165 | 54% | Confirmed cases |
|  | 2017 | 312 | 25% | Confirmed cases |
|  | 2018 | 633 | 27% | Confirmed cases |
|  | 2019 | 554 | 22% | Confirmed cases |
| Sierra Leone |  |  |  |  |
|  | 1996 | 246 | 30% | Not specified |
|  | 1997 | 470 | 23% | Not specified |
|  | 2004-2008 | 729 | 23% | Suspected cases |
| Guinea |  |  |  |  |
|  | 1996-1999 | 22 | 18% | Confirmed cases |
