# Supplementary material for: The number of cases, mortality and treatments of viral hemorrhagic fevers: A systematic review
Source: PLoS Negl Trop Dis. 2022 Oct 31;16(10):e0010889. doi: 10.1371/journal.pntd.0010889 (PMC9648854; doi:10.1371/journal.pntd.0010889)
Supplement: S11 Table — (DOCX) [file pntd.0010889.s012.docx]

S11 Table. Number of cases and CFRs of Marburg virus disease by country and period

| **Country** | **Period** | **Number of cases** | **Case fatality rate** | **Case definition** |
| --- | --- | --- | --- | --- |
| Angola |  |  |  |  |
|  | 2005 | 374 | 88% | Not specified |
| Democratic Republic of the Congo |  |  |  |  |
|  | 1998-2000 | 154 | 83% | Not specified |
| Germany |  |  |  |  |
|  | 1967 | 29 | 24% | Not specified |
| ex-Yougoslavia |  |  |  |  |
|  | 1967 | 2 | 0% | Not specified |
| South Africa |  |  |  |  |
|  | 1975 | 3 | 33% | Not specified |
| Kenya |  |  |  |  |
|  | 1980 | 2 | 50% | Not specified |
|  | 1987 | 1 | 100% | Not specified |
| Uganda |  |  |  |  |
|  | 2007 | 4 | 50% | Not specified |
|  | 2012 | 15 | 27% | Not specified |
|  | 2014 | 1 | 100% | Not specified |
|  | 2017 | 2 | 100% | Confirmed cases |
