# Supplementary material for: The number of cases, mortality and treatments of viral hemorrhagic fevers: A systematic review
Source: PLoS Negl Trop Dis. 2022 Oct 31;16(10):e0010889. doi: 10.1371/journal.pntd.0010889 (PMC9648854; doi:10.1371/journal.pntd.0010889)
Supplement: S12 Table — (DOCX) [file pntd.0010889.s013.docx]

S12 Table. Number of cases and CFRs of Rift Valley fever by country and period

| **Country** | **Period** | **Number of cases** | **Case fatality rate** | **Case definition** |
| --- | --- | --- | --- | --- |
| Egypt |  |  |  |  |
|  | 1977-1978 | 18000 | 3% | Not specified |
|  | 2003 | 148 | 18% | Not specified |
| Kenya |  |  |  |  |
|  | 1997-1998 | 46 | NR | Confirmed cases |
|  | 2006-2007 | 684 | 49% | Not specified |
|  | 2018 | 26 | 23% | Not specified |
| Madagascar |  |  |  |  |
|  | 2008-2009 | 712 | 4% | Suspected cases |
| Mauritania |  |  |  |  |
|  | 2010 | 63 | 21% | Not specified |
|  | 2012 | 36 | 50% | Not specified |
|  | 2015 | 31 | 26% | Not specified |
| Mayotte (France) |  |  |  |  |
|  | 2018-2019 | 129 | NR | Not specified |
| Mozambique |  |  |  |  |
|  | 2008 | 412 | 4% | Not specified |
| Niger |  |  |  |  |
|  | 2016 | 17 | 29% | Confirmed cases |
| Saudi Arabia |  |  |  |  |
|  | 2000-2001 | 683 | 14% | Confirmed cases |
| Somalia |  |  |  |  |
|  | 2006-2007 | 114 | 45% | Not specified |
| South Africa |  |  |  |  |
|  | 2008 | 17 | 0% | Confirmed cases |
|  | 2009 | 7 | 0% | Confirmed cases |
|  | 2010 | 241 | 10% | Confirmed cases |
|  | 2011 | 37 | 0% | Confirmed cases |
| Sudan |  |  |  |  |
|  | 2007-2008 | 747 | 31% | Confirmed cases |
| Tanzania |  |  |  |  |
|  | 2007 | 309 | 47% | Probable or confirmed cases |
| Yemen |  |  |  |  |
|  | 2000 | 1087 | 11% | Suspected cases |

*Note: NR, Not reported*
