# Supplementary material for: The number of cases, mortality and treatments of viral hemorrhagic fevers: A systematic review
Source: PLoS Negl Trop Dis. 2022 Oct 31;16(10):e0010889. doi: 10.1371/journal.pntd.0010889 (PMC9648854; doi:10.1371/journal.pntd.0010889)
Supplement: S13 Table — (DOCX) [file pntd.0010889.s014.docx]

S13 Table. Number of cases and CFRs of Alkhurma hemorrhagic fever, Argentine hemorrhagic fever, Bolivian hemorrhagic fever, Chapare hemorrhagic fever, Lujo hemorrhagic fever, Omsk hemorrhagic fever, Sabia hemorrhagic fever and Venezuelan hemorrhagic fever by country and period

| **Country** | **Period** | **Number of cases** | **Case fatality rate** | **Case definition** |
| --- | --- | --- | --- | --- |
| **Alkhurma hemorrhagic fever** | | | | |
| Saudi Arabia |  |  |  |  |
|  | 1994 – 2005 | 24 | 25% | Confirmed cases |
|  | 2009 – 2011 | 233 | 0.4% | Confirmed cases |
|  | 2003 – 2009 | 78 | 1% | Confirmed cases |
| **Argentine hemorrhagic fever** | | | | |
| Argentina |  |  |  |  |
|  | 1990 | 481 | NR | Confirmed cases |
|  | 1991 | 452 | NR | Confirmed cases |
|  | 2016 | 16 | NR | Confirmed cases |
|  | 2017 | 19 | NR | Confirmed cases |
|  | 2018 | 13 | NR | Confirmed cases |
| **Bolivian hemorrhagic fever** | | | | |
| Bolivia |  |  |  |  |
|  | 1959-1962 | 470 | 30% | Not specified |
|  | 2007 | 20 | 15% | Suspected cases |
|  | 2008 | 200 | 6% | Suspected cases |
| **Chapare hemorrhagic fever** | | | | |
| Bolivia |  |  |  |  |
|  | 2003 | NA | NR | Not specified |
|  | 2019 | 5 | 60% | Confirmed cases |
| **Lujo hemorrhagic fever** | | | | |
| South Africa |  |  |  |  |
|  | 2008 | 5 | 80% | Not specified |
| **Omsk hemorrhagic fever** | | | | |
| Russia |  |  |  |  |
|  | 1946-1958 | 972 | NR | Not specified |
|  | 1988-1997 | 165 | NR | Not specified |
|  | 1998 | 7 | 14% | Not specified |
| **Sabia hemorrhagic fever** | | | | |
| Brazil |  |  |  |  |
|  | 1990-2017 | 4 | 2% | Not specified |
| **Venezuelan hemorrhagic fever** | | | | |
| Venezuela |  |  |  |  |
|  | 1989-2010 | 728 | 23% | Not specified |

*Note: NR, Not reported*
