# Supplementary material for: The number of cases, mortality and treatments of viral hemorrhagic fevers: A systematic review
Source: PLoS Negl Trop Dis. 2022 Oct 31;16(10):e0010889. doi: 10.1371/journal.pntd.0010889 (PMC9648854; doi:10.1371/journal.pntd.0010889)
Supplement: S14 Table — (DOCX) [file pntd.0010889.s015.docx]

S14 Table. Description of the treatment and comparator groups of each included studies reporting results on VHF treatments

| **Study** | **RCT?** | **Treatment** | **Treatment details** | **Comparator** | **Treatment duration** |
| --- | --- | --- | --- | --- | --- |
| **Argentine hemorrhagic fever** | | | | | |
| **Maiztegui 1979** [1] | Yes | Immune plasma | 500mL intravenous immune plasma obtained from donors convalescent from AHF, in whom a four-fold or greater rise in antibody titres had been demonstrated with complement-fixation tests. | Normal plasma obtained from donors without a history of AHF who were residents of Buenos Aires, a city located outside the endemic area of the disease. | NA |
| **Crimean-Congo hemorrhagic fever** | | | | | |
| **Elaldi 2009** [2] | No | Ribavirin | Oral ribavirin (30 mg/kg initial loading dose, followed by 15 mg/kg every 6 h for 4 days, followed by 7.5 mg/kg every 8 h for 6 days). | Patients who were diagnosed in 2003 and did not receive ribavirin. | 10 days |
| **Dokuzoguz 2013** [3] | No | Ribavirin +/- Corticosteroid | Oral ribavirin 4 g daily for 4 days, followed by 2.4 g daily for 6 days Patients whose condition deteriorated rapidly despite receipt of ribavirin were given 10 mg/m2 dexamethasone. | No treatment | 10 days |
| **Cevik 2008** [4] | No | Ribavirin | Intravenous ribavirin 17mg/kg loading dose then 17 mg/kg every 6h for 4 days and then 8 mg/kg every 8h for 6 days + Supportive treatment | No ribavirin | 10 days |
| **Yilmaz 2016** [5] | No | Ribavirin | NR | Non-ribavirin | NR |
| **Izadi 2009** [6] | No | Ribavirin | For adults, 2 g of ribavirin had been prescribed initially as a loading dose, followed by 1 g every 6 h for 4 days and then 500 mg every 8 h for 6 days.  For children, a 30 mg/kg bolus was initially administered, followed by 15 mg/kg every 6 h for 4 days, and 7.5 mg/kg every 8 h for the next 6 days. | NR | 10 days |
| **Tulek 2012** [7] | No | Ribavirin | NR | Patients with CCHF of the other infectious diseases department (same hospital) who didn't receive ribavirin | Minimum 48h of treatment |
| **Koksal 2010** [8] | Yes | Ribavirin | Oral ribavirin (30 mg/kg initial loading dose, followed by 15 mg/kg every 6 h for 4 days, followed by 7.5 mg/kg every 8 h for 6 days). | Only supportive therapy without ribavirin | 10 days |
| **Mardani 2003** [9] | No | Ribavirin | Oral ribavirin (30 mg/kg initial loading dose, followed by 15 mg/kg every 6 h for 4 days, followed by 7.5 mg/kg every 8 h for 6 days). | Historical controls not treated with ribavirin | 10 days |
| **Salehi 2013** [10] | Yes | Immune globulins (IGIV) + ribavirin | Intra-venousy, 30-50g of IGIV, in association with standard therapy of Ribavirin (30mg/kg as loading dose, then 15mg/kg QID for four days, and continued 8mg/kg TID for six days) | Only ribavirin as standard therapy | 10 days |
| **Ebola Virus Disease** | | | | | |
| **Aluisio 2019** [11] | No | Intravenous fluid (IVF) | NR | NR | Median duration of treatment was 4 (IQR, 3–6) days |
| **Aluisio 2019** [12]**; Aluisio 2019** [13] | No | Vitamin A supplementation | Oral vitamin A supplementation of a capsule formulation containing retinyl palmitate 100,000 IU. The IMC guidelines also recommended supplementation with ascorbic acid dosed 500 mg 3 times daily with ascorbic acid 250 mg tablets and multivitamin once daily. Two formulations of multivitamins were used. The first sourced included vitamin A (2500 IU), thiamin (1 mg), riboflavin (0.5 mg), niacin (7.5 mg), ascorbic acid (15 mg), and cholecalciferol (300 IU), while the second included vitamin A (800 IU), thiamin (0.5 mg), riboflavin (0.5 mg), niacin (7.5 mg), and cholecalciferol (200 IU). | No vitamin A supplementation | 2 days |
| **Aluisio 2020** [14] | No | Cephalosporin | Oral third-generation cephalosporin, Cefixime. The Cefixime protocol was 400 mg once daily for the first five days of ETU care. | No treatment | 5 days |
| **Bai 2016** [15] | No | Favipiravir + WHO-recommended therapies | WHO-recommended therapies + an oral dose of favipiravir (T-705): 800 mg taken twice in the first day (1600 mg total) and 2 doses of 600 mg on day 2, followed by at least 5 days of standard therapy | WHO-recommended schedules | 3-11 days |
| **Sissoko 2016** [16] | No | Favipiravir + Standard care | •Standard care included oral or intravenous rehydration, electrolyte correction, empiric antimalarial and antibacterial therapies, analgesics, and antiemetic drugs. •Oral Favipiravir. The adult dose was 6,000 mg on day 0 (first dose: 2,400 mg; second dose [8 h after the first dose]: 2,400 mg; third dose [8 h after the second dose]: 1,200 mg) and 2,400 mg (1,200 mg twice a day) on day 1 to day 9 | Historical data (540 patients hospitalized between 15 September and 15 December 2014, the 3 months preceding the start of the JIKI study) | 10 days |
| **Yam 2020** [17] | No | Multivitamin supplementation | Two primary multivitamin formulations were used based on availability. The first included Vitamin A (2500 IU), B1 (1 mg), B2 (0.5 mg), B3 (7.5 mg), C (15 mg), and D3 (300 IU), while the second included Vitamin A (800 IU), B1 (0.5 mg), B2 (0.5 mg), B3 (7.5 mg), and D3 (200 IU). In addition to the multivitamins, the guidelines also recommended Vitamin A (1000 retinol units on Day 1 and 2) and Vitamin C (500 mg three times daily) supplementation, though actual care varied based on supply availability. | Patients not treated with multivitamins | 48h |
| **Dunning 2016** [18] | No | Brincidofovir + supportive care | • Patients with bodyweight ≥50 kg at enrolment received 200 mg loading dose of oral brincidofovir on day 0, followed by 100 mg brincidofovir on days 3, 7, 10, and 14. Patients with bodyweight <50 kg received 4 mg/kg oral brincidofovir as an initial dose on day 0, followed by 2 mg/kg on days 3, 7, 10, and 14.  • Supportive care : including intravenous crystalloids or oral rehydration solution, antimicrobials, antimalarials, analgesics, and nutritional support as prescribed | NR | 14 days |
| **Gignoux 2015** [19] | No | • Artemether-Lumefantrine • Artesunate-Amodiaquine | • According to protocol, all patients with suspected EVD who were admitted to the Ebola treatment center were supposed to be prescribed standard treatment consisting of prophylactic antibiotics and a 3-day course of the antimalarial combination therapy artemether-lumefantrine, with the dose determined according to the age of the patient. • However, on August 19, 2014, the supply of artemether-lumefantrine ran out, and during the subsequent 12-day “stock-out” period, patients who would have normally been prescribed artemether-lumefantrine were prescribed a 3-day course of artesunate-amodiaquine, with the dose determined according to the age of the patient. | No Antimalarial Drug Prescription | 3 days |
| **Garbern 2019** [20] | No | Artesunate-amodiaquine (ASAQ) | All patients admitted to the ETUs received antimalarial treatment empirically (regardless of whether a malaria RDT was performed) with oral artemether-lumefantrine (89.6% of patients) or parenteral artemether (4.5% of patients) or artesunate (5.7% of patients) if unable to take oral medications, in accordance with recommended guidelines. Patients who were admitted to the ETUs during the time periods of ASAQ’s therapeutic effect and whose reported home residence was in a chiefdom that received the MDA were considered exposed to ASAQ. | Not exposed to ASAQ | NR |
| **Dunning 2016** [21] | No | TKM-130803 + standard supportive care | TKM-130803 was a liquid (non-lyophilised) formulation of siEbola-3 with LNP1. TKM-130803 was administered at a dose of 0.3 mg/kg/d for 7 d by intravenous infusion at a rate of 1.25 ml/min over 2 h, for a total infused volume of 150 ml. | NR | 7 days |
| **Sahr 2017** [22] | No | Convalescent whole blood (CWB) + routine care | • CWB was administered within the first 24 h of admission. One unit (450 ml) of ABO compatible blood was administered to consented subject over a period of one to four hours. The blood was obtained and tested by blood services technicians of the Sierra Leone Ministry of Health and Sanitation from 60 consented blood donors that had recovered from Ebola in Kenema Town, Eastern Sierra Leone. Donated blood was obtained after three months of recovery. • Routine care was given to all the patients; briefly, patients were given IV fluids, multivitamins, antipyretics, analgesics, antibiotics, anthelmintics and antimalarial drugs when deemed necessary. | Not treated with CWB | 1-4 hours |
| **Konde 2017** [23] | No | IFN-beta 1a | Subcutaneous injection daily with doses ranging from 60 to 510 micrograms depending on the patient | The historical control patients were admitted to the Coyah ETU during the same time period as the IFN beta-1a patients with RT-PCR confirmed blood EBOV. Also included 17 patients who matched the IFN treated patients for eligibility criteria based on <6 days from symptom onset, age, under care in a Guinean treatment centre, who were better matched for baseline CT values. | NA |
| **Sadek 1999** [24] | No | Whole blood transfusion from convalescent patients | NR | NR | NR |
| **van Griensven 2016** [25] | No | Transfusion of convalescent plasma | Two consecutive transfusions of 200 to 250mL of ABO-compatible convalescent plasma (400 to 500mL in total), with each unit of plasma obtained from a separate convalescent donor; small adults and children weighing less than 45kg received two transfusions of 10mL of convalescent plasma per kilogram of body weight. Each transfusion was administered over a 20minute period, with a 15min interval between the two transfusions | Patients who had been admitted to the ETU during the preparatory period of the study while the system for apheresis and pathogen reduction was being set up and those for whom ABO compatible convalescent plasma was not available during the study. At the start of recruitment, there was a sufficient amount of convalescent plasma available to treat all the patients, so a protocol amendment was approved for the control group to consist of patients who were treated at the same ETU before the initiation of the trial. | 1 day |
| **Davey 2016** [26] | Yes | Zmapp + supportive care | Mixture of 3 monoclonal antibodies directed against the surface glycoprotein of EBOV. Zmapp treatment was begun within 12 to 24 hours after randomization and consisted of 3 intravenous infusions of Zmapp (50mg per kilogram of body weight), administered every third day. | Standard of care | 10 days |
| **Mulangu 2019** [27] | Yes | •Remdesivir •Mab114  •REGN-EB3 | •Intravenous remdesevir with a loading dose on day 1 (200mg in adults, and adjusted for body weight in pediatric patients), followed by a daily maintenance dose (100mg in adults), starting on day 2 and continuing for 9 to 13 days, depending on viral load.  •Intravenous Mab114 with a dose of 50mg per kilogram, administered as a single infusion on day 1. •Intravenous REGN-EB3 with a dose of 150mg per kilogram, administered as a single infusion on day 1. | Zmapp (intravenous 50mg per kilogram of body weight every third day beginning on day 1 for a total of 3 doses) | Zmapp : 9 days ; Remdesevir : 9 to 13 days, REGN-EB3 and Mab114 : 1 day |
| **Kerber 2019** [28] | No | Favipiravir | Oral favipiravir on a compassionate-use basis. Loading dose of 6000mg on the first day followed by 2400mg/days for 9 days. | Non treated patients | 10 days |
| **Hantavirus Pulmonary Syndrome** | | | | | |
| **Chapman 1999** [29] | No | Ribavirin | From June to November 1993, intravenous ribavirin was initially used with a one-time loading dose of 30 mg/kg (up to a maximum of 2g) followed by 15 mg/kg every 6 h for 4 days, and then 7.5 mg/kg every 8 h for 6 days. From November 1993 to September 1994, the dosing schedule was modified to conform to that used for Hantaan virus-associated illness in China: a one-time loading dose of 33 mg/kg (up to a maximum of 2 g) followed in 6 h by 16 mg/kg (up to a maximum of 1 g) every 6 h for 15 doses, followed by 8 mg/kg (up to a maximum of 500 mg) every 8 h for nine doses. | Contemporaneous untreated HPS patients | 10 days |
| **Mertz 2004** [30] | Yes | Ribavirin | Intravenous ribavirin (33 mg/kg [≤2 g] as a loading dose, followed by 16 mg/kg [≤1 g] given q6h for 4 days and by 8 mg/kg [≤500 mg] given q8h for 3 days) | Placebo | 7 days |
| **Vial 2013** [31] | Yes | Methylprednisolone | 8 mg/kg (up to 500 mg) in 100 cc D5W (or in 50 cc for children <20 kg) by intravenous infusion over 1 hour followed by the same dose administered over 23 hours. On days 2 and 3, 16 mg/kg (up to 1000 mg) methylprednisolone was diluted in 200 cc D5W (100 cc for children <20 kg) and administered over 24 hours. | Placebo | 3 days |
| **Wernly 2011** [32] | No | Extracorporeal membrane oxygenation (ECMO) support | Patients who had elective insertion of vascular sheaths and were almost concurrently intubated and placed on ECMO when they decompensated. | Patients intubated when they became hypoxic and placed on ECMO when they became hemodynamically unstable | NA |
| **Vial 2015** [33] | No | Immune plasma infusion | Dose of 5,000 units of Nab per kg of body weight; in patients subjected to ECMO, a second 5,000 units/kg dose was administered. | Untreated patients | 1 day |
| **Hemorrhagic fever with renal syndrome** | | | | | |
| **Gui 1987** [34] | Yes | Recombinant interferon α-2 | Each patient was treated with interferon for 5 days beginning on the day of admission. Ten patients received 20 x 10^6 units im each day for 3 days and then 10 x 10^6 units for 2 days. The total amount received during the 5-day treatment was 80 x 10^6 units. The remaining 15 patients received 10 x 10^6 units daily for 5 days, for a total of 50 x 10^6 units. | Placebo | 5 days |
| **Du 2013** [35] | No | Renal Replacement Therapy (RRT) | RRT including continuous RRT (CRRT) and intermittent hemodialysis (IHD) | No RRT | NR |
| **Huggins 1991** [36] | Yes | Ribavirin | Ribavirin iv in a loading dose of 33 mg/kg, followed by a dose of 16 mg/kg every 6 h for the first 4 days and 8 mg/kg every 8 h for the subsequent 3 days (modified from 6 days after experience with 21 patients in 1985-1986) | Placebo | 7 days |
| **Lassa fever** | | | | | |
| **Ilori 2019** [37] | No | Ribavirin | NR | Not ribavirin | NA |
| **McCormick 1986** [38] | Yes | PHASE 1 : No therapy VS Oral Ribavirin VS Lassa convalescent plasma PHASE 2 : intravenous ribavirin +/- lassa plasma | PHASE 1 Oral ribavirin: in a 3g loading dose followed by 1g per day in divided doses everyeight hours for 10 days. // Lassa convalescent plasma: received 1 unit (approximately 4mL per kg of body weight) with an immunofluorescent titer >1:128. PHASE 2: intravenous ribavirin, 2g loading dose and 1g every six hours for four days, reduced to 0.5 given intravenously every eight hours for another 6 days. // same ribavirin regimen with 1 unit of 300mL of convalescent plasma given with the first dose of ribavirin | No therapy | Ribavirin oral : 10 days // Lassa plasma : 1 day // Ribavirin IV : 10 days // Rivarin + plasma : 10 days |

*Note: RCT, randomized controlled trial; NR, Not reported; NA, Not applicable*

References

1. Maiztegui JI, Fernandez NJ, de Damilano AJ. Efficacy of immune plasma in treatment of Argentine haemorrhagic fever and association between treatment and a late neurological syndrome. Lancet Lond Engl. 1979 Dec 8;2(8154):1216–7.

2. Elaldi N, Bodur H, Ascioglu S, Celikbas A, Ozkurt Z, Vahaboglu H, et al. Efficacy of oral ribavirin treatment in Crimean-Congo haemorrhagic fever: a quasi-experimental study from Turkey. J Infect. 2009 Mar;58(3):238–44.

3. Dokuzoguz B, Celikbas AK, Gök ŞE, Baykam N, Eroglu MN, Ergönül Ö. Severity scoring index for Crimean-Congo hemorrhagic fever and the impact of ribavirin and corticosteroids on fatality. Clin Infect Dis Off Publ Infect Dis Soc Am. 2013 Nov;57(9):1270–4.

4. Cevik MA, Elaldi N, Akinci E, Ongürü P, Erbay A, Buzgan T, et al. A preliminary study to evaluate the effect of intravenous ribavirin treatment on survival rates in Crimean-Congo hemorrhagic fever. J Infect. 2008 Oct;57(4):350–1.

5. Yilmaz G., Sunbul M., Yapar D., Baykam N., Hasanoglu I., Guner R., et al. Ribavirin in treatment of crimean-congo hemorrhagic fever (CCHF): An international multicenter retrospective analysis. Open Forum Infect Dis. 2016;3.

6. Izadi S, Salehi M, S. I, M. S. Evaluation of the efficacy of ribavirin therapy on survival of Crimean-Congo hemorrhagic fever patients: A case-control study. Jpn J Infect Dis. 2009 Jan;62(1):11–5.

7. Tulek N., Ozturk B., Bulut C., Tuncer Ertem G., Erdinc F.S., Altun S., et al. The evaluation of ribavirin use in patients with Crimean-Congo haemorrhagic fever. Clin Microbiol Infect. 2012;18:579–80.

8. Koksal I, Yilmaz G, Aksoy F, Aydin H, Yavuz I, Iskender S, et al. The efficacy of ribavirin in the treatment of Crimean-Congo hemorrhagic fever in Eastern Black Sea region in Turkey. J Clin Virol. 2010 Jan;47(1):65–8.

9. Mardani M, Jahromi MK, Naieni KH, Zeinali M. The efficacy of oral ribavirin in the treatment of crimean-congo hemorrhagic fever in Iran. Clin Infect Dis Off Publ Infect Dis Soc Am. 2003 Jun 15;36(12):1613–8.

10. Salehi H, Salehi MM, Adibi N, Salehi MM, H. S, M. S, et al. Comparative study between Ribavirin and Ribavirin plus Intravenous Immunoglobulin against Crimean Congo hemorrhagic fever. J Res Med Sci. 2013 Jun;18(6):497–500.

11. Aluisio A.R., Yam D., Peters J.L., Cho D.K., Perera S.M., Kennedy S.B., et al. Impact of Intravenous Fluid Therapy on Survival Among Patients with Ebola Virus Disease: An International Multisite Retrospective Cohort Study. Clin Infect Dis Off Publ Infect Dis Soc Am. 2019;ciz344.

12. Aluisio AR, Perera SM, Yam D, Garbern S, Peters JL, Abel L, et al. Vitamin A Supplementation Was Associated with Reduced Mortality in Patients with Ebola Virus Disease during the West African Outbreak. J Nutr. 2019 Oct 1;149(10):1757–65.

13. Aluisio A.R., Yam D., Peters J., Cho D., Perera S., Kennedy S., et al. Association between vitamin a supplementation and mortality in Ebola virus disease: A multisite cohort study. Acad Emerg Med. 2019;26:S67.

14. Aluisio A.R., Perera S.M., Yam D., Garbern S., Peters J.L., Abel L., et al. Association Between Treatment with Oral Third-Generation Cephalosporin Antibiotics and Mortality Outcomes in Ebola Virus Disease: A Multinational Retrospective Cohort Study. Trop Med Int Health TM IH. 2020;10.1111/tmi.13369.

15. Bai C.-Q., Mu J.-S., Kargbo D., Song Y.-B., Niu W.-K., Nie W.-M., et al. Clinical and Virological Characteristics of Ebola Virus Disease Patients Treated with Favipiravir (T-705) - Sierra Leone, 2014. Clin Infect Dis. 2016;63(10):1288–94.

16. Sissoko D, Laouenan C, Folkesson E, M’Lebing AB, Beavogui AH, Baize S, et al. Experimental Treatment with Favipiravir for Ebola Virus Disease (the JIKI Trial): A Historically Controlled, Single-Arm Proof-of-Concept Trial in Guinea. PLoS Med. 2016 Mar 1;13(3):e1001967–e1001967.

17. Yam D., Aluisio A.R., Perera S.M., Peters J.L., Cho D.K., Kennedy S.B., et al. Association between multivitamin supplementation and mortality among patients with Ebola virus disease: An international multisite cohort study. Afr J Emerg Med. 2020;23–9.

18. Dunning J, Kennedy SB, Antierens A, Whitehead J, Ciglenecki I, Carson G, et al. Experimental Treatment of Ebola Virus Disease with Brincidofovir. PloS One. 2016 Sep 9;11(9):e0162199–e0162199.

19. Gignoux E., Azman A.S., Ciglenecki I. Artesunate-amodiaquine is associated with reduced ebola mortality. Am J Trop Med Hyg. 2015;93(4):446.

20. Garbern SC, Yam D, Aluisio AR, Cho DK, Kennedy SB, Massaquoi M, et al. Effect of Mass Artesunate-Amodiaquine Distribution on Mortality of Patients With Ebola Virus Disease During West African Outbreak. Open Forum Infect Dis. 2019 May 24;6(7):ofz250–ofz250.

21. Dunning J, Sahr F, Rojek A, Gannon F, Carson G, Idriss B, et al. Experimental Treatment of Ebola Virus Disease with TKM-130803: A Single-Arm Phase 2 Clinical Trial. PLoS Med. 2016 Apr 19;13(4):e1001997–e1001997.

22. Sahr F, Ansumana R, Massaquoi TA, Idriss BR, Sesay FR, Lamin JM, et al. Evaluation of convalescent whole blood for treating Ebola Virus Disease in Freetown, Sierra Leone. J Infect. 2017 Mar;74(3):302–9.

23. Konde MK, Baker DP, Traore FA, Sow MS, Camara A, Barry AA, et al. Interferon β-1a for the treatment of Ebola virus disease: A historically controlled, single-arm proof-of-concept trial. PloS One. 2017 Feb 22;12(2):e0169255–e0169255.

24. Sadek RF, Khan AS, Stevens G, Peters CJ, Ksiazek TG. Ebola hemorrhagic fever, Democratic Republic of the Congo, 1995: determinants of survival. J Infect Dis. 1999 Feb;179 Suppl 1:S24–7.

25. van Griensven J, Edwards T, de Lamballerie X, Semple MG, Gallian P, Baize S, et al. Evaluation of Convalescent Plasma for Ebola Virus Disease in Guinea. N Engl J Med. 2016 Jan 7;374(1):33–42.

26. Davey R, Dodd L, Proschan M, Neaton J, Neuhaus Nordwall J, Koopmeiners J, et al. A Randomized, Controlled Trial of ZMapp for Ebola Virus Infection. N Engl J Med. 2016;375(15):1448‐1456.

27. Mulangu S, Dodd LE, Davey RT Jr, Tshiani Mbaya O, Proschan M, Mukadi D, et al. A Randomized, Controlled Trial of Ebola Virus Disease Therapeutics. N Engl J Med. 2019 Dec 12;381(24):2293–303.

28. Kerber R, Lorenz E, Duraffour S, Sissoko D, Rudolf M, Jaeger A, et al. Laboratory Findings, Compassionate Use of Favipiravir, and Outcome in Patients With Ebola Virus Disease, Guinea, 2015-A Retrospective Observational Study. J Infect Dis. 2019 Jun 19;220(2):195–202.

29. Chapman LE, Mertz GJ, Peters CJ, Jolson HM, Khan AS, Ksiazek TG, et al. Intravenous ribavirin for hantavirus pulmonary syndrome: safety and tolerance during 1 year of open-label experience. Ribavirin Study Group. Antivir Ther. 1999;4(4):211–9.

30. Mertz GJ, Miedzinski L, Goade D, Pavia AT, Hjelle B, Hansbarger CO, et al. Placebo-controlled, double-blind trial of intravenous ribavirin for the treatment of hantavirus cardiopulmonary syndrome in North America. Clin Infect Dis Off Publ Infect Dis Soc Am. 2004 Nov 1;39(9):1307–13.

31. Vial PA, Valdivieso F, Ferres M, Riquelme R, Rioseco ML, Calvo M, et al. High-dose intravenous methylprednisolone for hantavirus cardiopulmonary syndrome in Chile: a double-blind, randomized controlled clinical trial. Clin Infect Dis Off Publ Infect Dis Soc Am. 2013 Oct;57(7):943–51.

32. Wernly JA, Dietl CA, Tabe CE, Pett SB, Crandall C, Milligan K, et al. Extracorporeal membrane oxygenation support improves survival of patients with Hantavirus cardiopulmonary syndrome refractory to medical treatment. Eur J Cardio-Thorac Surg Off J Eur Assoc Cardio-Thorac Surg. 2011 Dec;40(6):1334–40.

33. Vial P.A., Valdivieso F., Calvo M., Rioseco M.L., Riquelme R., Araneda A., et al. A non-randomized multicentre trial of human immune plasma for treatment of hantavirus cardiopulmonary syndrome caused by Andes virus. Antivir Ther. 2015;20(4):377–86.

34. Gui X, Ho M, Cohen M, Wang Q, Huang H, Xie Q. Hemorrhagic fever with renal syndrome: treatment with recombinant alpha interferon. J Infect Dis. 1987;155(5):1047‐1051.

35. Du H., Li J., Yu H.-T., Jia Z.-S., Yu D.-H., Wang J.-P., et al. The optimal timing of RRT for critical patients with hemorrhagic fever with renal syndrome. Ther Apher Dial. 2013;17(5):A2.

36. Huggins JW, Hsiang CM, Cosgriff TM, Guang MY, Smith JI, Wu ZO, et al. Prospective, double-blind, concurrent, placebo-controlled clinical trial of intravenous ribavirin therapy of hemorrhagic fever with renal syndrome. J Infect Dis. 1991 Dec;164(6):1119–27.

37. Ilori E.A., Furuse Y., Ipadeola O.B., Dan-Nwafor C.C., Abubakar A., Womi-Eteng O.E., et al. Epidemiologic and clinical features of lassa fever outbreak in Nigeria, january 1-may 6, 2018. Emerg Infect Dis. 2019;25(6):1066–74.

38. McCormick JB, King IJ, Webb PA, Scribner CL, Craven RB, Johnson KM, et al. Lassa fever. Effective therapy with ribavirin. N Engl J Med. 1986 Jan 2;314(1):20–6.
