# Supplementary material for: The number of cases, mortality and treatments of viral hemorrhagic fevers: A systematic review
Source: PLoS Negl Trop Dis. 2022 Oct 31;16(10):e0010889. doi: 10.1371/journal.pntd.0010889 (PMC9648854; doi:10.1371/journal.pntd.0010889)
Supplement: Attachment — Submitted filename: MS_Response_Reviewers_20220805.docx [file pntd.0010889.s016.docx]

Drifa Belhadi

INSERM IAME UMR 1137 – Université Paris Cité

16 rue Henri Huchard

75018 Paris, France

E-Mail: drifa.belhadi@aphp.fr

Paris, August 5^th^, 2022

MS ID: PNTD-D-22-00729

Dear Editors,

We thank you and both referees for the detailed and constructive comments on our manuscript entitled “The number of cases, mortality and treatments of viral hemorrhagic fevers: a systematic review” by Drifa Belhadi, Majda El Baied, Guillaume Mulier, Denis Malvy, France Mentré, Cédric Laouénan, for publication in PLOS Neglected Tropical Diseases.

The points mentioned are quite relevant. Most of these aspects have been added in the manuscript. You will find below a point-by-point response to the comments and description of the changes made in the manuscript.

Thank you for the attention you will pay to our revised manuscript.

Sincerely yours,

Drifa Belhadi

**Editor’s comments:**

***Please review the figures, including Fig 1 (Flowchart) making sure that the numbers add up and including the number of duplicates removed at each stage for each group. For example: 57+96=153 =! 141 in Panel A.***

We thank you for your comment. We added the number of duplicates removed in Fig 1.

***Please also explain or clarify whether the Case fatality rate (CFR) was estimated by the authors of primary studies or if was instead estimated by the authors of this review using the derived information and how.***

We agree that the information was missing in the manuscript. The following sentences were added in the “Methods” section:

*“Numbers were extracted as reported in each study. If the CFR was not reported in a study, the number of cases and the number of deaths were used, if available, to estimate the missing CFR.”*

--------------------

**Methods**

**Reviewer #1: *Methodology:***

***-Were the references of the articles included reviewed?***

***- The researchers reviewed review articles and their references, for example:***

***N Engl J Med 2020; 382:1832-1842***

We did not manually reviewed the references of the articles due to the high number of included studies and the heterogeneity of studies published in this topic. We clarified this in the “Methods” section:

*“Reference lists of included studies were not reviewed.”*

**Reviewer #2:** ***The methods have been clearly explained.***

We thank you for your comment.

--------------------

**Results**

**Reviewer #1:** ***Results:***

***Line 205 - Place the abbreviations on line 102***

The abbreviations reported on line 205 (line 211 in the revised manuscript) correspond to the figure 2 caption. We also reported the abbreviations in the text on line 102.

***Line 374 – remind the reader that dengue and yellow fever were excluded***

We modified the sentence to make it clearer:

“To our knowledge, this is the first comprehensive systematic review to summarize all published information available on worldwide cases numbers, mortality and treatments of a range of VHFs, *excluding severe dengue and yellow fever*.”

***Tables:***

***-Table 2 – In the “Comparator” column adjust the descriptions. For example, one box says 'No ribavirin' (Cevik 2008, Yilmaz 2016) and another box says 'Only supportive therapy without ribavirin' (Koksal 2010). Does this mean that those who did not receive ribavirin also did not receive supportive treatment?***

***Is “NR” (Izadi 2009) the same as “No treatment” (Dokuzoguz 2013) or “No vitamin A supplementation” (Aluisio 2019 -408, 313)?***

***Same question for “No antimalarial drug prescription, Not exposed to ASAQ, Not treated with CWB, NA, etc.***

We modified and clarified the treatment and comparator columns in Table 2 in order to report more explicitly the information present in each article.

***-Clarify abbreviations: NR, NA (always place them at the foot of the tables)***

We harmonized the abbreviations and added footnotes to clarify them.

***Figures:***

***Check the resolution quality of the figures***

We checked the figures to make sure all of them follow the requirement of the journal (300 – 600 dpi).

**Reviewer #2:** ***The authors have done well in presenting their results***.

***The author has taken an onerous task of carrying analyses and summarizing many studies on diseases occurring in such diverse circumstances. Despite being informative It risks diluting or oversimplifying results of such a heterogeneous group of diseases taking places in difference eras and circumstances. For example, Line 191: Did the authors look at whether there was a difference in the quality of studies between studies carried out in earlier years compared to later years? That should be mentioned. Other factors such as CFR can also be affected by the improvement in technology used as supportive in addition to medicinal treatments.***

Indeed the date of publication may have an impact on the quality of the studies. Therefore, we added a comparison of studies published before or after 2010 in the “Risk of bias of included studies” section:

“On the 38 studies reporting results on treatments, 10 were randomized trials and 28 were non-randomized. Regarding the randomized comparative studies, only two were classified as having a “low risk of bias”, *both published after 2010,* and the other eight studies were classified as having “some concerns” (cf. S3 Table in Supplementary Material). Regarding the non-randomized studies, 13 were classified as “poor quality”, 5 as “fair quality” and 10 as “good quality” (cf. S4 Table in Supplementary Material). *On those 28 non-randomized studies, 6 were published before 2010 and 67% of them (n=4/6) were classified as “poor quality”, compared with 41% (n=9/22) for those published after 2010.*

The 88 studies reporting number of cases and CFR were evaluated using the Newcastle-Ottawa Quality Assessment Form: 59 were classified as “poor quality” and 29 as “fair quality” (cf. S5 Table in Supplementary Material). *On these 88 studies, 31 were published before 2010 and a similar proportion than those published after 2010 were classified as “poor quality” (65%, n=20/31, for studies before 2010, compared with 68%, n=45/66, for studies published after 2010)*.”

--------------------

**Conclusions**

**Reviewer #1:** ***The conclusions are clear***

We thank you for your review.

**Reviewer #2:** ***It should be noted in the conclusion that treatment facilities in some places in Africa where treatment for VHF cases was taking place in the during period of outbreak, may not be ideal and can account for some higher CFR.***

Indeed, care can vary across country, especially in Africa and can explain high mortality rates. We added this element to the “Discussion” section.

*“Mortality rates varied also greatly across countries, especially in Africa, which can explained by the fact that treatment facilities in some places in Africa may be limited.*”

***It is important for the authors to make reference to some important inherent weaknesses of the study context as described by the authors of each study that has been included in the analysis.***

More details were added to the discussion on the limitations associated with the study context reported in included studies:

“*The study context also has an impact on the results of our review. Some authors report the challenge they can face of studying a relatively rare disease that affect widely dispersed rural areas.[41] Regarding VHFs such as EVD, authors reported difficulties during the course of their study, with for example interruption of participating centers due to violence from local community or paramilitary groups who can be suspicious of the activities in those facilities.[37]*”

--------------------

Editorial and Data Presentation Modifications?

**Reviewer #1:** ***Minor Revision***

**Reviewer #2:** ***Accept***

--------------------

**Summary and General Comments**

**Reviewer #1:** ***This study aimed to carry out a systematic review of all reports and clinical studies that included specific results on the number of cases, mortality, and treatments of viral hemorrhagic fevers (VHF), focused mainly on BSL-4 pathogens (excluded dengue and yellow fever).***

***Results from the last two decades are presented, where limited therapeutic options are observed.***

***This results in a call to action for decision-makers to be aware of the existing gap and propose new studies.***

***Advantage:***

***-The revision is registered in PROSPERO***

***Disadvantages/Limitations:***

***-The databases included were MEDLINE, Embase, and CENTRAL***

***-Gray literature (e.g., OpenGrey) and data on ClinicalTrials.gov were not included***

***-Limited to English and French language (add this information in the methods section)***

******Consider adding these limitations in the discussion section (line 393)***

The language restriction was added to the “Methods” section:

**“*Study language****. Included studies were limited to studies published in English or French.”*

The following limitation was added to the “Discussion” section:

“*Moreover, we restricted our review of the grey literature to national health websites and references reported on the WHO and CDC websites and data on clinical trials registries (e.g. ClinicalTrials.gov) were not included here.*”

**Reviewer #2:** ***(No Response)***

--------------------

**PLOS authors have the option to publish the peer review history of their article (what does this mean?). If published, this will include your full peer review and any attached files.**

**Reviewer #1:** Yes: Luis Gabriel Parra-Lara

**Reviewer #2:** No
