# Supplementary material for: The number of cases, mortality and treatments of viral hemorrhagic fevers: A systematic review
Source: PLoS Negl Trop Dis. 2022 Oct 31;16(10):e0010889. doi: 10.1371/journal.pntd.0010889 (PMC9648854; doi:10.1371/journal.pntd.0010889)
Supplement: Attachment — Submitted filename: MS_Response_Second_Reviewers_20221004.docx [file pntd.0010889.s017.docx]

Drifa Belhadi

INSERM IAME UMR 1137 – Université Paris Cité

16 rue Henri Huchard

75018 Paris, France

E-Mail: drifa.belhadi@aphp.fr

Paris, October 4^th^, 2022

MS ID: PNTD-D-22-00729

Dear Editors,

We thank you and the referees for the detailed and constructive comments on our manuscript entitled “The number of cases, mortality and treatments of viral hemorrhagic fevers: a systematic review” by Drifa Belhadi, Majda El Baied, Guillaume Mulier, Denis Malvy, France Mentré, Cédric Laouénan, for publication in PLOS Neglected Tropical Diseases.

The points mentioned are quite relevant. Most of these aspects have been added in the manuscript. You will find below a point-by-point response to the comments and description of the changes made in the manuscript.

Thank you for the attention you will pay to our revised manuscript.

Sincerely yours,

Drifa Belhadi

**Reviewer comments:** ***Note: I was asked to review this manuscript after it had already been through a round of peer review.***

***The stated goal of the manuscript was to review CFR, geography and treatment of "VHFs". There is a lack of precision in the term "VHF". The authors exclude two of the most globally relevant VHFs: YF and Dengue, yet include new world hantaviruses and LCMV, which are associated with pulmonary syndrome and encephalitis respectively and don't exhibit prominent hemorrhagic symptoms. They also state that they are looking at "BSL-4" VHFs but the Hantaviruses, LCMV and RVFV are NOT BSL-4 viruses. Some clarity is needed regarding which pathogens are selected for study and why. Maybe since there really wasn't any data for the tickborne flavi's, you just exclude them and state that you are looking at selected bunyas, filos and arenas? this is c/w the ones you chose for narrative description and figures.***

We selected the VHFs based on the list from the CDC website that we accessed when we wrote our protocol in December 2019 and subsequently did not include severe dengue and yellow fever, though we acknowledge the two conditions may be otherwise considered. Indeed, we should not use the “BSL-4” term as it does not fit with the classification of some selected VHFs. Hence, we reviewed our wording in order to make it less confusing:

Lines 71-73: “*The agents that are causative of VHF are often classified as Biosafety Level 4 (BSL-4) pathogens that require special laboratory facilities with the highest level of safety measures.”*

Lines 91-93: “*We decided to focus mostly on VHFs caused by a selection of arenaviridae, filoviridae, bunyaviridae and flaviviridae and did not look at some other important VHF conditions such as the severe dengue and yellow fever.”*

***Table 3: I assume HR is Hazard Ratio, but this is not defined in the table.***

Indeed, we clarified all the acronyms that were used in the table.

***How can a study have an outcome result and a p value when the outcome has no measure? eg Dokuzokguz 2013 corticosteroids +/- ribavirin vs no treatment- all parameters are listed as NR (ie not reported, according to the table).***

We thank you for your comment. Indeed, there was an error in our reporting of the results in the table. We corrected the table accordingly.

***Dunning 2016-1888 (what does 1888 here mean?): how can you have an outcome in the comparator group but no N in the comparator group?***

Indeed “1888” was referring to an internal reference system. We removed it from the table. Regarding the Dunning 2016 publication, the sample size of the comparator group was not reported as the mortality rate was associated with general patients’ data from the 2014-2015 Ebola virus disease outbreak. We added in the table the total number of patients and the description of the patients used to obtain this mortality rate.

***Some mention should be made, especially for the Ebola treatment data that it only applies to one strain of virus (ie Zaire, rather than Sudan, Bundi, etc). Same for HPS- are these studies from patients with Andes? SNV? a different New World Hanta? Lassa from different regions also has very divergent phylogeny, so results are not generalizable to all disease.***

We thank you for your relevant comment. More details were added to the discussion:

Lines 408-419: *“It is also important to mention that we did not stratify our results according to species or the strain of the virus. For example, in the case of Ebola disease investigational therapeutic options, the trials of importance were conducted during the 2014-2016 western Africa outbreak and the 2018-2020 North-Kivu (Democratic Republic of the Congo) outbreak that were related to Ebola virus (species Zaire Ebolavirus) and Makona and Kikwit specific strain respectively [36, 37]. With respect to treatment of other ebolavirus diseases (e.g. Soudan and Bundibugyo virus diseases), options are even more limited. Notably, the three monoclonal antibody treatments tested in the PALM trial [37], ZMapp, REGN-EB3, and MAb114, have a narrow spectrum and are ineffective against other filovirus infections. In the case of other VHFs such as HPS or LF, the sparse comparative trials assessed mainly a nucleoside inhibitor (i.e., ribavirin). No strong proof of effectiveness of ribavirin was identified for HPS. Regarding LF, the efficacy of ribavirin was not considered as specific to Lassa virus lineage. Besides, considerable uncertainty was recently even more raised about its activity as an anti-infectious agent in the management of the condition. [52-54]”*
